# A novel population of extracellular vesicles smaller than exosomes promotes cell proliferation

**DOI:** 10.1186/s12964-019-0401-z

**Published:** 2019-08-15

**Authors:** Sang-Soo Lee, Jong-Hoon Won, Gippeum J. Lim, Jeongran Han, Ji Youn Lee, Kyung-Ok Cho, Young-Kyung Bae

**Affiliations:** 1 Department of Biological Sciences, Korea Advanced Institute of Science and Technology, 291 Daehak-ro, Yuseong-gu, Daejeon, Korea; 2 Center for Bioanalysis, Korea Research Institute of Standards and Science, 267 Gajeong-ro, Yuseong-gu, Daejeon, Korea

**Keywords:** Extracellular vesicles, Exosomes, Cell proliferation, P200 fraction, CD81

## Abstract

**Background:**

Extracellular vesicles (EVs) play important roles in intercellular communication by delivering RNA, lipid, and proteins to neighboring or distant cells. Identification and classification of EVs secreted from diverse cell types are essential for understanding their signaling properties.

**Methods:**

In this study, EVs from the culture media were isolated by ultracentrifugation and analyzed by electron microscopy (EM) and nanoparticle tracking analyses. Conditioned media (CM) from HEK293 cells culture grown either in serum-free (SF) or 10% fetal bovine serum (FBS) containing media were centrifuged at 100,000×g to separate the SN_Δ_ supernatant and the P100 pellet in which exosomes are enriched. Then, the SN_Δ_ fraction was centrifuged at 200,000×g to yield the P200 pellet fraction containing novel EVs smaller than exosomes. The exosomal markers in the EV subgroups were examined by western blotting and immune-EM, and the functional analyses of EVs were conducted on HEK293 and THP-1 cell culture.

**Results:**

We identified a new group of EVs in the P200 fraction that was smaller than exosomes in size. Typical exosome markers such as Hsp70, TSG101, and CD63 were found in both P100 exosomes and the P200 vesicles, but CD81 was highly enriched in exosomes but not in the P200 vesicles. Furthermore, chemicals that inhibit the major exosome production pathway did not decrease the level of P200 vesicles. Therefore, these small EVs indeed belong to a distinguished group of EVs. Exosomes and the P200 vesicles were found in CM of human cell lines as well as FBS. Addition of the exosomes and the P200 vesicles to human cell cultures enhanced exosome production and cell proliferation, respectively.

**Conclusions:**

Our study identifies a novel population of EVs present in the P200 fraction. This EV population is distinguished from exosomes in size, protein contents, and biogenesis pathway. Furthermore, exosomes promote their own production whereas the P200 vesicles support cell proliferation. In sum, we report a new group of EVs that are distinct physically, biologically and functionally from exosomes.

**Electronic supplementary material:**

The online version of this article (10.1186/s12964-019-0401-z) contains supplementary material, which is available to authorized users.

## Background

Intercellular communication plays a pivotal role in all multicellular organisms, and membrane-bound extracellular vesicles (EVs) are essential components for modulating the cellular functions of neighboring and remote cells by delivering multiple components such as proteins, RNA, and lipids [1–3]. Such EVs have been identified from almost all mammalian cell types including stem cells [4–6], immune cells [7, 8], and the primary cells of the nervous system [9, 10] as well as numerous cancer cell lines [11, 12]. In addition, these vesicles can easily be separated from most body fluids such as serum, plasma, urine, and cerebrospinal fluid [1, 2]. Therefore, studies on these EVs are important not only for understanding their biological roles but also for exploiting their functions in medical applications.

Various types of EVs such as exosomes, micro-vesicles, and smaller vesicles differ widely in their sizes and biogenesis [13–15]. Exosomes are generally 40–100 nm in diameter originated from fusion of multi-vesicular body (MVB) to plasma membrane [16, 17]. It has been shown that signaling molecules such as Wnt and Hedgehog are secreted by exosomal secretion pathway [18–23], indicating exosomes and perhaps other types of EVs play an important role in signaling. Several proteins have been conventionally used to mark MVB and exosomes. Exosome markers include members from the tetraspanin family (e.g. CD9, CD63 and CD81), components of the ESCRT complex (e.g. TSG101 and Hsp70), and the Rab family (e.g. Rab7 and Rab9) [16, 18, 24]. Micro-vesicles (100–1,000 nm) are generated by budding from the plasma membrane [25], whereas smaller vesicles (20–50 nm) have unclear origin [15, 26]. Despite their distinct origins, size ranges of these EVs overlap considerably and their proper classification and nomenclature are still being developed.

**Fig. 1:**
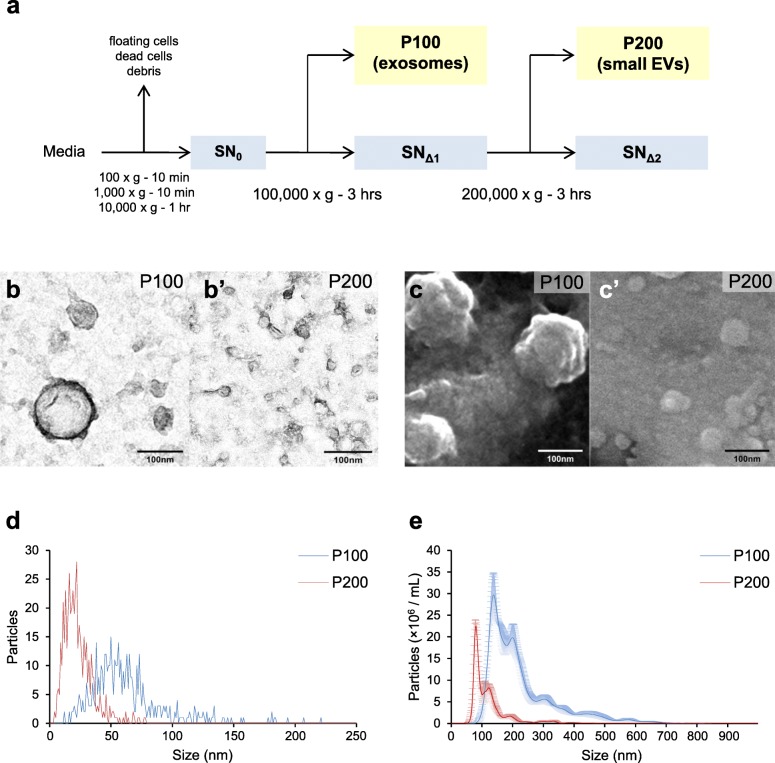
Identification of vesicles in the P200 fraction. (**a**) Experimental scheme for serial centrifugations to isolate sub-fractions (**b**,**b’**) Representative TEM images of (**b**) the P100 and (**b’**) P200 fraction from HEK293 CM. (**c**,**c’**) Representative ESEM images of (**c**) the P100 and (**c’**) P200 fractions from HEK293CM. (**d**) Histograms for the size distribution of particles in the P100 (blue) and P200 (red) fractions from TEM images of HEK293 CM in (b,b’) (*n* = 500). (**e**) Histograms for the size distribution and concentrations of particles in the P100 (blue) and P200 (red) fractions analyzed by NTA. Scale bar: 100 nm

Ultracentrifugation is one of the most common methods to isolate exosomes, which aims to obtain vesicles within a specific size range [19, 27, 28]. To obtain exosomes, the conditioned media (CM) are cleared to remove floating or dead cells by a series of low spins, and the cleared media (SN_0_) are then centrifuged at 100,000×g to separate the supernatant SN_Δ_ fraction and the pellet P100 fraction in which exosomes are enriched (Fig. 1a).

Here, we identified a new type of EVs in the P200 pellet fraction by a 200,000×g centrifugation of the remaining supernatant fraction after exosome isolation (Fig. 1a). We used biochemical assays, NTA, and EM to determine the distinct characteristics of small EVs in the P200 fraction. More importantly, we found that vesicles in P100 (exosomes) and P200 (small EVs) fractions are capable of enhancing distinct cellular functions: P100 induces exosome production and P200 supports cell proliferation.

## Methods

### Cell culture

HEK293 and THP-1 cells were grown in RPMI 1640 media (Gibco) supplemented with 10% FBS (Gibco) or exosome depleted FBS (ED-FBS, EXO-FBS, SBI) at 37 °C, 5% CO_2_. *Drosophila* S2 cells were grown in M3 media (Sigma-Aldrich) supplemented with 10% IMS (Sigma-Aldrich) at 25 °C.

### Western analysis and antibodies

For western analysis, samples were mixed with the 5 × SDS sample buffer and boiled at 95 °C for 10 min. Samples were then separated by 10% SDS-PAGE gel (Mini-PROTEAN TGXTM Gels, Bio-Rad) and transferred to nitrocellulose membrane. Membranes were blocked with 5% nonfat milk in TBST buffer (Intron), and probed with a primary antibody. After washing membranes with TBST four times, membranes were incubated with horseradish peroxidase (HRP)-conjugated secondary antibody in TBST with 5% nonfat milk. After washing, protein bands were visualized using the ECL system (Millipore).

Following antibody dilutions were used for western analysis: Hsp70 (SBI, rabbit), 1:1000~1500; TSG101 (SBI, rabbit), 1:1000; CD63 (SBI, rabbit), 1:1000; CD81 (SBI, rabbit), 1:500~1,000; CD81 (Santa Cruz, mouse), 1:200; HRP (SBI, rabbit or mouse), 1:10,000.

### Exosome isolation

For the ultracentrifugation method, conditioned media (12 mL) were first centrifuged at 100×g for 10 min, and the supernatant is then centrifuged at 1,000×g for 10 min. The resulting supernatant was centrifuged at 10,000×g to remove relatively large particles such as cell debris at 4 °C. Using a Beckman Coulter Optima L-90 K ultracentrifuge with a type 41Ti rotor, the cleared supernatant SN_0_ is spun down at 100,000×g for 3 h at 4 °C to obtain the exosome fraction in the pellet (P100) and the supernatant fraction SN_Δ1_ [19]. SN_Δ1_ was subsequently spun down at 200,000×g for 3 h, yielding the pellet P200 and the supernatant SN_Δ2_. To remove contaminants, the P100 and P200 fractions were resuspended with 5 mL of PBS and then spun down with 100,000×g and 200,000×g for 3 h at 4 °C, respectively. For all applications including western blots analyses, NTA, and EM, the pellets obtained from the indicated volume of media were resuspended in 60 μL PBS. SN_Δ2_ was concentrated in Amicon® Ultra-4 10 kDa nominal molecular weight centrifugal filter units to a final volume of 60 μL.

For qEV Size Exclusion Columns (iZON) method, samples were prepared according to the manufacturers’ instructions. Briefly, 12 mL conditioned media were concentrated in Amicon® Ultra-4 10 kDa nominal molecular weight centrifugal filter units to a final volume of 1 mL. Then, this concentrated sample was overlaid on a prepared qEV column, eluted with PBS and sequentially collected every 0.5 mL. Each fraction was then concentrated to a final volume of 60 μL for western analysis. The particle number and protein concentration were determined with Nanosight (NS300 or LM10, Malvern) and the Bradford assay (Bio-Rad), respectively. Exoquick-TC™ (System Biosciences) was used according to the manufacturer’s instructions.

### BFA and GW4869 treatments

Brefeldin A (BFA, Sigma) and N-SMase inhibitor (GW4869, Sigma) dissolved in DMSO were used at 10 μg / mL and the same (v/v) concentration of DMSO was used for control. When the HEK293 cell culture reached ~ 80% confluency in T75 flasks, the cells were treated with BFA in serum-free (SF) media for 15 min after PBS washing. Treated cells were then washed again with PBS and cultured in SF media for 2 h. Treatment with GW4869 was same as above except the final culture time in SF was 1 h. To check whether these treatments have any effects on the vesicles that have been already secreted to media, pellets obtained from 2 mL of HEK293 CM by Exo-quick were resuspended in 2 mL PBS and incubated with either BFA or GW4869 (10 μg / mL) for 1 h.

### Treatment of cell cultures with ultracentrifugal sub-fractions

12 mL of fresh 10% FBS media, 10% ED-FBS media, or HEK293 CM (72 h, either in 10% FBS or 10% ED-FBS, 24 h, in SF) was used to obtain the following sub-fractions (SN_0_, SN_Δ1_, SN_Δ2_, SN_Δ2_ + P100, P100, P200) with the ultracentrifuge method for all experiments, except for the time-course experiments in Additional file 4: Figure S4e, f where 24 mL of original media was processed. For treating cells with P100 and P200, P100 or P200 pellets were thoroughly dissolved in SF media before supplied to THP-1 or HEK293 cells. For treating cells with supernatant fractions, 2 mL of either SN_0_, SN_Δ1_, SN_Δ2_ or SN_Δ2_ + P100 was supplied to THP-1 or HEK293 cells. Liposomes (Plain Pure DOPC Liposomes (100 nm), FormuMax) were used at the concentration of 0.1 μg / mL in all experiments. The initial cell number for THP-1 and HEK293 cells were 1.25 × 10^5^ per mL in all experiments, except for the time-course experiments in Additional file 4: Figure S4e, f where 2.5 × 10^5^ cells per mL was used in 6 well plates. After 48 h, the exosomes were isolated by Exo-quick from each sample medium and measured by NTA using a NanoSight (NS300 or LM10, Malvern). In Additional file 5: Figure S5 g, the P200 from each sample medium was isolated by ultracentrifugation in T75 flask. The number of cells was counted using Countess (Invitrogen) and the viability was analyzed by trypan blue dye exclusion assays.

### Electron microscopy

For transmission electron microscopy (TEM), samples were prepared using the Exosome-TEM-easy Kit that contains a Formvar-carbon coated EM mesh 400 grid, wash buffer, and EM solution (101 Bio). The P100 and P200 pellets from 12 mL media, were resuspended in 60 μL PBS. SN_0,_ SN_Δ1,_ and SN_Δ2_ were concentrated in Amicon® Ultra-4 10 kDa nominal molecular weight centrifugal filter units to a final volume of 60 μL, from which 10 μL was applied on the grid. Samples were prepared according to the manufacturer’s instruction. For immuno-EM, the pellets isolated from 36 mL media were first fixed with 60 μL of 4% paraformaldehyde fixation buffer (BioLegend) + 0.2% glutaraldehyde (Sigma) for 30 min. Fixed exosome solution was then transferred to the grid, and the grid was treated with 0.05 M glycine for 10 min to quench free aldehyde groups. After blocking with PBS containing 1% BSA for 30 min, the grid was incubated for 1 h with anti-CD63 antibody (SBI) (diluted 1:100 in PBS containing 0.1% BSA) at room temperature. After several washes with PBS containing 0.1% BSA, the grid was incubated for 1 h with the secondary antibody (anti-rabbit IgG conjugated to 10 nm gold particle, 1:25, Sigma) at room temperature. After several washes to remove secondary antibody, incubation in EM solution and washing were followed. The samples were viewed using Talos F200X transmission electron microscope (FEI) operated at 200 kV, and images were captured with a Ceta 16 M pixel CMOS camera (FEI). The size of vesicles was measured using Image J software for 500 vesicles from multiple TEM images. For environmental scanning electron microscopy (ESEM), the pellets were first fixed with 4% paraformaldehyde. After 1/8 dilution with PBS, 10 μL was added to the SEM holder and dried for 24 h. The ESEM holder was custom made with high purity copper to hold the sample and platinum was sputtered to sample on the holder before imaging. The ESEM images were acquired using the Quanta FEG 650 (FEI) environmental scanning electron microscope operated at 30 kV and FEI software.

### Nanoparticle tracking analysis

**Fig. 2:**
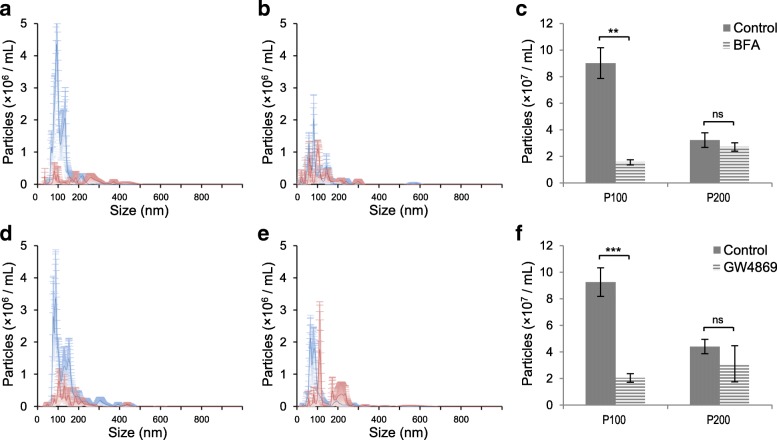
Inhibitiors of exosome production inhibit secretion of P100 vesicles but not P200 vesicles. **a**, **b** The histograms show (**a**) the numbers of particles in the P100 fractions and (**b**) those in the P200 fractions after treatment with BFA (red) and control (blue). **c** The bar graphs show the number of vesicles in the P100 and P200 fractions isolated from HEK293 cell CM after BFA treatment (shaded) and control (solid). **d**, **e** The histograms show (**d**) particles in the P100 fractions and (**e**) particles in the P200 fractions after treatment with GW4869 (red) and control (blue). **f** The bar graphs show the number of vesicles in the P100 and P200 fractions from GW4869 treated HEK293 CM (shaded) and control (solid). Values = mean ± S.D. *Significant difference analyzed by t-test (*p < 0.05,**p < 0.01,***p < 0.001)

For all applications including NTA, the P100 and P200 pellets were resuspended in 60 μL PBS. SN_0,_ SN_Δ1,_ and SN_Δ2_ were concentrated in Amicon® Ultra-4 10 kDa nominal molecular weight centrifugal filter units to a final volume of 60 μL. 10 μL of each prepared fraction was diluted to 1:100 in PBS. When pellet was obtained from 2 mL of media with Exo-quick, the pellet was resuspended in 1 mL of PBS for NTA. Eventually, vesicles originated from 2 mL media were concentrated to 1 mL of PBS and this dilution factor was taken into account when calculating particle concentrations. The number of particles per mL media was calculated using the corresponding dilution. Samples were analyzed by NTA using the NanoSight NS300 (Malvern) (Figs. 1, 2, 4g, h, 5g, h, 6b, d, Additional file 1: Figure S1, Additional file 3: Figure S3a-d, i-l, Additional file 4: Figure S4, Additional file 5: Figure S5) or LM10 (Malvern) (4f, h, 5f, h, 6e, f, Additional file 3: Figure S3e-h, Additional file 7: Figure S7), each equipped with a 405 nm laser. Videos were collected and analyzed using the NTA software (version 3.1, Malvern). To properly represent vesicles of different sizes, detection threshold settings were 5 (P100) or 2 (P200) [29]. The number of vesicles for each sample is presented as the number of particles per mL media (mean ± S.D., *n* = 6).

### Statistical analyses

Experiments were independently repeated at least in triplicates (*n* = 3 or 6), and data generated from six NTA results (mean ± S.D., n = 6) were analyzed by the Welch’s t-test (two-tailed) Microsoft Excel 2013 (Microsoft). Error bars in the graphical data represent means ± S.E.M. The Statistical significance was claimed when the *p*-value was lower than 0.05.

## Results

### Vesicles identified in the P200 fraction are smaller than exosomes

Numerous previous studies have considered the P100 fraction obtained from ultracentrifugation at 100,000×g as an exosome fraction [19, 27, 28, 30]. However, it is unknown whether there are any unidentified groups of vesicles in the supernatant SN_Δ_ after 100,000×g centrifugation [19]. To examine if this supernatant fraction (hereafter named as SN_Δ1_) has any EVs, the SN_Δ1_ was subsequently centrifuged at 200,000×g to obtain the P200 pellet and SN_Δ2_ supernatant fractions (Fig. 1a).

We then directly visualized the vesicles in the P100 and P200 fractions prepared from HEK293 cell culture media, using transmission electron microscopy (TEM) and environmental scanning electron microscopy (ESEM). The TEM micrographs of the P100 fraction showed vesicles with diameters ranging between 20 and 150 nm (Fig. 1b, d). On the other hand, the vesicles in the P200 fraction were smaller than those in the P100 fraction (Fig. 1b’,d). The size difference of the vesicles in the P100 and P200 fractions was also apparent in ESEM images (Fig. 1c,c’). Similar results were obtained with the CM of *Drosophila* S2 cells (Additional file 1: Figure S1c,c’,d), suggesting secretion of these smaller P200 vesicles is evolutionarily conserved. We also confirmed that the SN_Δ1_ contains the vesicles whose sizes are similar to P200 vesicles but not the larger P100 vesicles (Additional file 1: Figure S1a’,b’). No visible vesicles were found in the SN_Δ2_ fraction (Additional file 1: Figure S1a”,b”) and in the pellet fraction after re-centrifugation of SN_Δ2_ at 200,000×g (Additional file 1: Figure S1a”’,b”’). These results suggest that CM contains no other smaller vesicles than P200 vesicles.

We further examined vesicles in the P100 and P200 fractions by nanoparticle tracking analysis (NTA). The P100 and P200 fractions had vesicles with clearly different sizes (Fig. 1e), reminiscing results obtained by EM. The diameter of the vesicles at the major peak was 138.3 nm in the P100 and 80.9 nm in the P200 fraction. Size measurement by both NTA and EM clearly demonstrates that the P200 fraction contains vesicles smaller than exosomes, although vesicle sizes obtained from TEM images were smaller than those by NTA as previously reported [31]. In addition, the NTA data confirmed that no vesicles are present in the SN_Δ2_ and in the pellet obtained from centrifugation of SN_Δ2_ at 200,000×g (Additional file 1: Figure S1e).

### The biogenesis of vesicles in the P200 fraction is different from exosomes

Multiple types of EVs are generated by distinct mechanisms [15], and exosomes are released to the extracellular space by fusion of MVB with the plasma membrane [16, 32–35]. A number of chemicals, such as Brefeldin A (BFA) and GW4869, have been reported to block a major step in exosome secretion [36–38]. BFA inhibits the guanine nucleotide-exchange protein BIG2 that regulates the constitutive release of exosome-like vesicles [39, 40]. GW4869 is neutral sphingomyelinase (N-SMase) inhibitor that negatively acts on the ceramide-mediated inward budding of MVB and release of mature exosomes [37, 41].

We hypothesized that the P200 vesicles are produced by a distinct mechanism rather than simply representing smaller exosomes. Therefore, we tested if these inhibitors of exosome secretion specifically block the secretion of vesicles in the P100 but not P200. BFA treatment to HEK293 cells decreased the number of particles in the P100 fraction to 1/10th compared to no treatment (Fig. 2a, c). A comparable result was obtained when HEK293 cells were treated with GW4869 (Fig. 2d, f). On the contrary, neither BFA nor GW4869 treatment significantly decreased the number of vesicles in the P200 fraction (Fig. 2b, c, e, f). BFA and GW4869 treatment changed neither the number nor the size of the vesicles that had been already secreted to the media, indicating that these drugs intracellularly affect biogenesis and secretion of exosomes but not the secreted extracellular exosomes (Additional file 1: Figure S1f, g). These results show that the BIG2 and N-SMase-dependent pathway is the major pathway by which P100 but not the P200 vesicles are produced. Therefore, vesicles in the P100 and P200 fractions are distinguishable by not only size but also their major biogenesis pathway.

### CD81 is highly enriched in exosomes but not in P200 vesicles

**Fig. 3:**
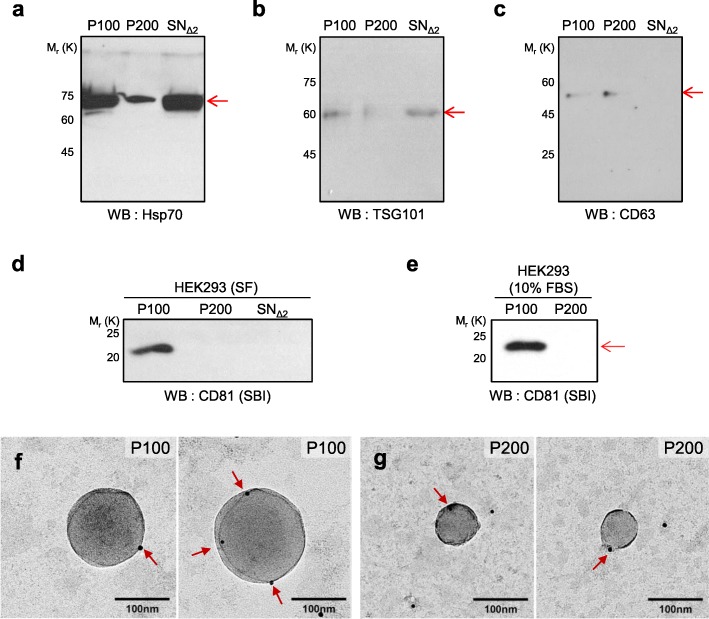
P100 and P200 fractions contain the different levels of exosome markers. **a-d** Sub-fractions were prepared from CM of HEK293 cells grown in SF condition for 72 h by ultracentrifuation and used for western blots to check the following exosome markers: (**a**) Hsp70, (**b**) TSG101, (**c**) CD63, and (**d**) CD81 (SBI, rabbit). Red arrow indicates the expected target protein size in each panel. **e** Western blot of the P100 and P200 fractions from CM of HEK293 cultured in 10% FBS media using anti-CD81 antibody. Red arrow indicates the CD81 protein. **f**, **g** Representative TEM images of the vesicles in (**f**) P100 and (**g**) P200 from the HEK293 CM labeled with 10 nm gold particles resulted from staining with an anti-CD63 antibody and anti-rabbit gold conjugated antibody. Red arrows indicate the CD63 protein. Original full blots are presented in Additional file 8: Figure S8a-e

We reasoned that some exosome markers among Hsp70, TSG101, CD63, and CD81 may not be present in P200 if smaller EVs are distinct from exosomes [16, 18, 24]. To test this, we prepared P100, P200, and SN_Δ2_ fractions from the CM of HEK293 cells by ultracentrifugation. When media containing 10% FBS were used for culture, the concentrated SN_Δ2_ samples failed to enter SDS-PAGE gels, presumably due to its high content of serum proteins. To circumvent this problem, HEK293 cells were cultured in SF media, and then the CM was harvested after 24 h. We found that Hsp70 and TSG101 were present in the P100, P200, and SN_Δ2_ fractions (Fig. 3a, b, Additional file 8: Figure S8a, b). CD63 was found in both P100 and P200 fractions, and the immuno-EM results confirmed that CD63 is present on the membrane structure of P100 and P200 vesicles (Fig. 3c, f, g, Additional file 2: Figure S2 h, i). On the other hand, CD63 was not detected in SN_Δ2_ fraction (Fig. 3c, Additional file 8: Figure S8c). This seems to reflect the nature of CD63 as a transmembrane protein that is likely associated with vesicular structure present in P100 and P200.

Presence or absence of these exosome markers in P100, P200, and SN_Δ2_ fractions prepared by ultracentrifugation was similar to those prepared by qEV size exclusion column (Additional file 2: Figure S2a-c, f-g). As the larger exosomes separate out first from the size exclusion column, the 7th to 9th fractions are known to contain exosomes. The smaller EVs appeared to be eluted in the 10th to 12th fractions, preceding the large quantity of soluble proteins in the later fractions (Additional file 2: Figure S2a). Hsp70 was present in all the qEV fractions after the 7th fraction, whereas CD63 was only detected in the 7th to 12th fractions (Additional file 2: Figure S2b, c, Additional file 9: Figure S9a, b). These two independent methods show similar results on the distribution of these exosome markers in different fractions.

Our survey with multiple exosome markers revealed that CD81 is highly enriched in the P100 fraction (i.e., exosomes) but not in the P200 (i.e., small EVs) and SN_Δ2_ (i.e., soluble proteins) fractions, using two different anti-CD81 antibodies (Fig. 3d, e; Additional file 2: Figure S2d, Additional file 9: Figure S9c). Additionally, CD81 was enriched in P100 but not in P200 fraction prepared from the CM of human monocytic leukemia cell line THP-1 (Additional file 2: Figure S2e, Additional file 9: Figure S9d). Enrichment of CD81 in the P100 fraction may be cell line specific, but this trend was repeatedly observed for HEK293 and THP-1 cell lines. CD81-positive bands were also consistently detected in 7th to 9th qEV fractions obtained from CM of HEK293 cells grown in either SF media or 10% FBS media (Additional file 2: Figure S2f, g, Additional file 9: Figure S9e, f), empathizing that CD81 is enriched in larger vesicles than P200 vesicles. We concluded that the smaller EVs are indeed a newly identified group of EVs distinct from exosomes, and CD81 is enriched specifically in exosomes but not in the small vesicles (i.e., P200) present in the CM of HEK293 cell culture.

### The P100 fraction promotes exosome production, while the P200 fraction supports cell proliferation

**Fig. 4:**
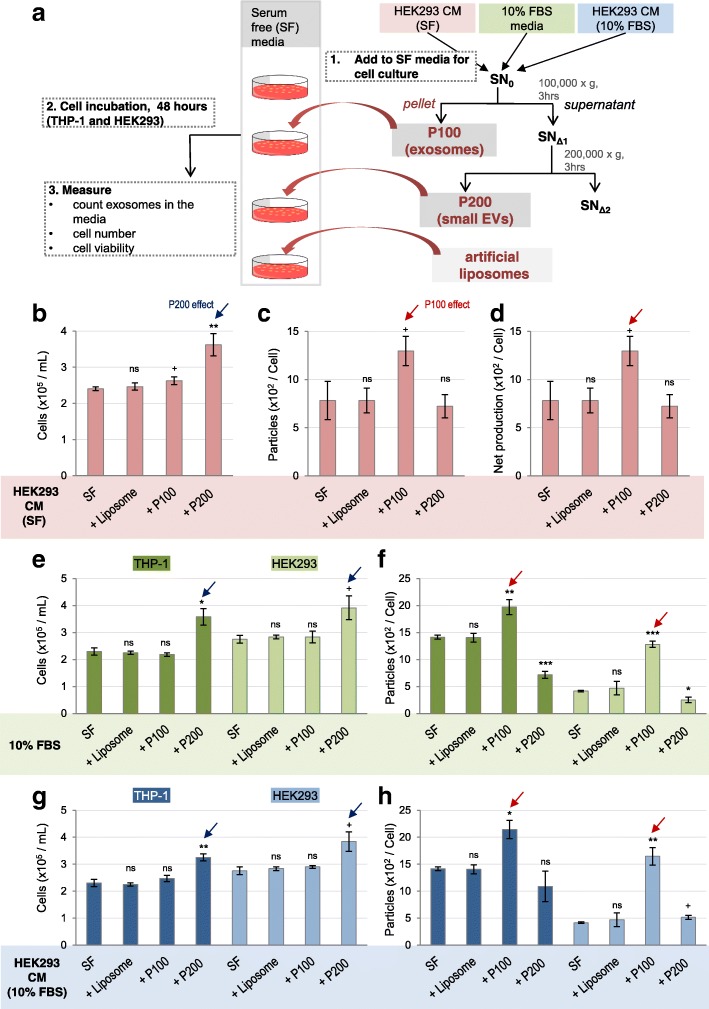
P100 fraction promotes exosome secretion while P200 fraction promotes cell proliferation. **a** A scheme to show experimental procedure. The fresh SF media, SF + P100 and SF + P200 fractions were supplied to the same number of THP-1 cells and HEK293 cells. After 48 h culture, the number of exosomes and cells were counted. **b-d** P100 and P200 fractions were prepared from HEK293 CM (SF), and were added to SF media. Liposomes were added as a negative control. The bar graphs show (**b**) the final cell numbers, (**c**) the number of exosomes per cell, and (**d**) the net production of exosomes per cell (EV_48h_ – EV_0h_) of HEK293 cell culture after each addition. (**e**, **f**) HEK293 cells were treated with the fractions prepared from 10% FBS. The bar graphs show (**e**) final cell numbers of THP-1 (dark green) and HEK293 cells (light green), and (**f**) the number of exosome normalized to the final cell numbers. **g, h** THP-1 or HEK293 cells were treated with the fractions prepared from HEK293 CM cultured in 10% FBS. The bar graphs show (**g**) the final cell numbers for THP-1 (dark blue) and HEK293 cells (light blue), and (**h**) the number of exosomes normalized to the final cell numbers. Red and blue arrows indicate marked effects by the P100 and the P200 fractions, respectively. Experiments were repeated three or six times, each including six technical repeats. The results are displayed as mean ± S.E.M. *Significant difference analyzed by t-test (*p < 0.05,**p < 0.01,***p < 0.001). ^+^ *p* < 0.1 and ns = not significant. SF = serum-free

Next, we asked if P100 and P200 fractions can elicit distinct cellular functions when supplied to cell culture media. We first prepared the P100 and P200 fractions from the HEK293 CM cultured in SF media to specifically obtain vesicles secreted from HEK293 cells because FBS contains bovine exosomes [42]. We dissolved these pellet fractions in SF media and measured the number of exosomes by NTA before supplying them to cell culture in order to obtain EV_0h_. Artificial liposomes were used as a negative control. After 48 h, numbers of cells and exosomes (EV_48h_) in the media were measured (Fig. 4a). We found that the cell numbers were significantly increased by addition of P200 but not by any other additions (Fig. 4b, blue arrows), suggesting that P200 is capable of promoting cell proliferation.

In the case of exosome production, supplying the P100 but not P200 from HEK293 cells considerably increased the number of released exosomes per cell (EV_48h_ per cell) compared to SF controls (Fig. 4c; red arrow). Likewise, the exosome numbers per mL media were increased only by the P100 addition (Additional file 3: Figure S3a). By subtracting the number of exosomes that are initially supplied (EV_0h_ per cell) from EV_48h_, we obtained the net EV production (EV_48h_ - EV_0h_). This value was substantially increased by adding P100 but not P200 (Fig. 4d; Additional file 3: Figure S3b). On the contrary, the P200 net production remained unaffected by addition of P200 vesicles (Additional file 5: Figure S5 g). This suggests that the P200 addition does not promote the production of P200. In sum, extracellular addition of exosomes from HEK293 cells promote extra production of exosomes far more than what HEK293 cells release without exosomes in SF controls (Fig. 4c, d).

It is known that FBS contains non-negligible amount of endogenous bovine exosomes [42]. We tested if FBS can promote additional exosome production from human cells. NTA confirmed that there are 1 ~ 10 × 10^8^ particles per mL in the P100 fraction prepared from fresh 10% FBS media (Additional file 4: Figure S4d, Additional file 5: Figure S5b). The number of exosomes slightly varied between FBS lots and preparation methods (data not shown). When HEK293 and THP-1 cells were grown in 10% FBS for 48 h, both exosome number per mL media and per cell sharply increased compared to SF conditions (Additional file 4: Figure S4a-c). The net increase in the number of exosomes in HEK293 CM grown in 10% FBS media for 48 h was far greater than the number of initially provided exosomes in fresh 10% FBS media (Additional file 4: Figure S4d; compare two adjacent shaded red and solid). These data suggest that bovine exosomes in FBS can induce exosome production from human cells.

We specifically examined if P100 exosomes and P200 small vesicles originated from FBS can elicit exosome production and cell proliferation, respectively, of two human cell lines, HEK293 and THP-1. Two different sets of P100 and P200 fractions were prepared to address this question: one set from FBS and the other from the CM of HEK293 cultured in 10% FBS media. We found that the P200 fractions from both sets markedly increase cell proliferation compared to SF control for both cell lines (Fig. 4e, g, blue arrows). On the other hand, the P100 fractions from both sets positively affected exosome production (Fig. 4f, h, Additional file 3: Figure S3e, g, red arrows). Cell viability of THP-1 and HEK293 cell lines was not affected by these treatments (Additional file 6: Figure S6a, b). In conclusion, the exosome fractions from both FBS and HEK293 CM (10% FBS and SF) promote exosome production, whereas the P200 fractions support cell proliferation of two human cell lines.

One interesting finding to note is that the bovine-origin P100 and P200 fractions can function in human cell lines. We further tested the inter-species effect with *Drosophila* S2 cell line. When the P100 and P200 fractions from HEK293 CM were added to artificial serum-containing media for S2 cell culture, we did not observe any changes in exosome release, cell proliferation, or viability (Additional file 3: Figure S3i-l).

To better understand the effects of FBS and its bovine exosomes on cells, cells were cultured in various concentrations (0, 2.5, 5, 10%) of FBS for 48 h. NTA accurately measured the number of exosomes in these fresh FBS media (EV_0h_) that proportionally increased to the concentration of FBS (Additional file 5: Figure S5b). Each of these FBS media (0, 2.5, 5, 10%) was supplied to HEK293 cells, and then the number of exosomes in CM were measured after 48 h (EV_48h_) (Additional file 5: Figure S5c, e). The calculated net production (EV_48h_ - EV_0h_ per cell and per mL) clearly demonstrates that FBS in media enhances exosome production of cells (Additional file 5: Figure S5, d, f). As expected, FBS supports cell proliferation (Additional file 5: Figure S5a). These combined data demonstrate that FBS containing bovine exosomes enhance production of exosomes from human cells.

We reasoned that bovine exosomes may be taken up by human cells in order to affect their exosome secretion. To test this, we cultured HEK293 and THP-1 cells in fresh SF media and in SF media plus the P100 fraction obtained from FBS. Then we collected these CM and measured the number of vesicles in P100 from each CM at six different time points (0 = before contact with cells, 1, 2, 6, 24, 48 h after culture). In SF condition as a control, the number of exosomes steadily increased during 1 to 6 h culture (Additional file 4: Figure S4e, f; gray dotted line), probably due to secretion of new exosomes. In contrary, in SF plus P100 condition, the number of exosomes initially decreased in an hour but increased over time (Additional file 4: Figure S4e, f; red dotted line). Such rapid internalization of extracellular exosomes has been previously reported [43, 44]. Interestingly, artificial liposomes, of which size and concentration matched with the P100 counterpart, exhibited somewhat dampened initial decrease and ultimately failed to induce the net increase in exosome production (Additional file 4: Figure S4e, f; orange dotted line). These data suggest that the provided bovine exosomes are taken up by the human cells within an hour, and in turn stimulate the exosome production.

### Depletion of P100 and P200 reduces exosome production and cell proliferation, respectively

**Fig. 5:**
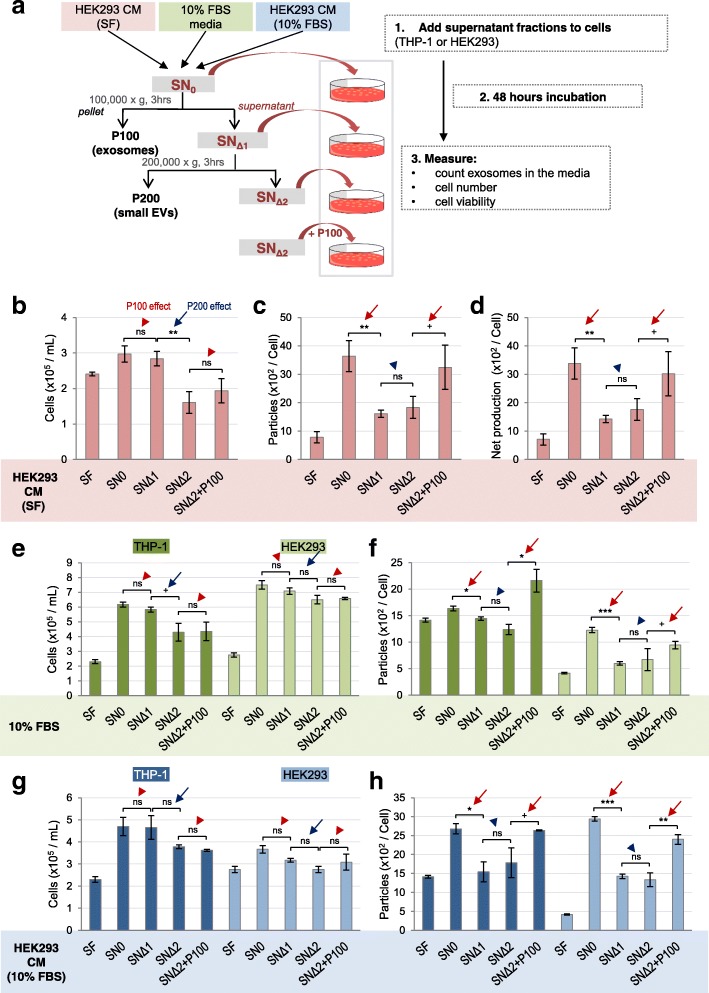
Depletion of P100 fraction reduces exosome production while depletion of P200 fraction reduces cell proliferation. **a** A scheme to show experimental procedures. SN_0_, SN_Δ1_, SN_Δ2_ and SN_Δ2_ + P100 fractions prepared from HEK293 CM and 10% FBS culture media were supplied to either THP-1 or HEK293 cell culture. **b-d** When HEK293 cells were treated with the fractions prepared from HEK293 CM cultured in SF. The bar graphs show (**b**) the final cell numbers, (**c**) the number of exosomes per cell, and (**d**) the net production of exosomes (EV_48h_ – EV_0h_). **e**, **f** HEK293 cells were treated with the fractions prepared from HEK293 CM cultured in 10% FBS. The bar graphs show (**e**) the final cell number and (**f**) the number of exosomes per final cell number for THP-1 (dark green) and HEK293 cells (light green). **g**, **h** HEK293 cells were treated with the fractions prepared from HEK293 CM cultured in 10% FBS. The bar graphs show (**g**) the final cell numbers and (**h**) exosome number per cell of THP-1 (dark blue) and HEK293 cells (light blue). Red and blue arrows indicate insignificant effects of the P100 and the significant effects of the P200 addition, respectively. Experiments were repeated three or six times, each including six technical repeats. The results are displayed as mean ± S.E.M. *Significant difference analyzed by t-test (*p < 0.05,**p < 0.01,***p < 0.001). ^+^ *p* < 0.1 and ns = not significant

In order to confirm our findings on differential functions of the two distinct EV groups, we designed a set of complementary experiments. Instead of the P100 and P200 pellet fractions, we prepared three supernatant fractions: SN_0_, SN_Δ1_ (=SN_0_-P100), and SN_Δ2_ (=SN_Δ1_-P200) from HEK293 CM (SF) (Fig. 5a). Additionally, the P100 fraction isolated from HEK293 CM (SF) was supplemented to the corresponding SN_Δ2_ to generate “SN_Δ2_ + P100”. Then, each of these was directly applied to HEK293 cells and the numbers of cells and exosomes in media were measured after 48 h culture (Fig. 5a). We found that the final cell number was significantly reduced in SN_Δ2_ compared to SN_Δ1_ (Fig. 5b, blue arrow). On the other hand, the differences in cell number between SN_0_ versus SN_Δ1_ and those between SN_Δ2_ versus SN_Δ2_ + P100 remained unchanged (Fig. 5b; red arrowheads). Consistent results were obtained when 10% FBS and HEK293 CM (10% FBS) supernatant fractions were added to THP-1 and HEK293 cell cultures (Fig. 5e, g, blue arrows). Thus, the depletion of P200 in SN_Δ2_ fraction, compared to the depletion of P100 in SN_Δ1_ fraction, hampered cell proliferation.

Next, we examined effects of the aforementioned fractions on exosome production. After 48 h of culture in SN_0_ from HEK293 CM (SF), HEK293 cells produced a large number of exosomes compared to SF controls. The exosome production per cell (EV_48h_) was decreased in SN_Δ1_ that lacks P100 compared to SN_0_, but increased in SN_Δ2_ + P100 compared to SN_Δ2_ (Fig. 5c; red arrows). SN_Δ1_ and SN_Δ2_ fractions did not show significant differences in production of exosomes, indicating that depletion of P200 does not affect exosome production (Fig. 5c; blue arrowheads). Similar results were obtained when the net production (EV_48h_ – EV_0h_) (Fig. 5d; red arrows) and the number of exosomes per mL (Additional file 3: Figure S3c, d) were considered. When THP-1 and HEK293 cell cultures were treated with fractions from 10% FBS and HEK293 CM (10% FBS), similar results were obtained with comparable cell viability (Fig. 5f, h; red arrows and blue arrowheads, Additional file 3: Figure S3f, h; Additional file 6: Figure S6c, d). Therefore, exosomes, the component depleted in SN_Δ1_ compared to SN_0_, contribute to exosome production.

### Exosome-depleted FBS promotes cell proliferation but not exosome production

**Fig. 6:**
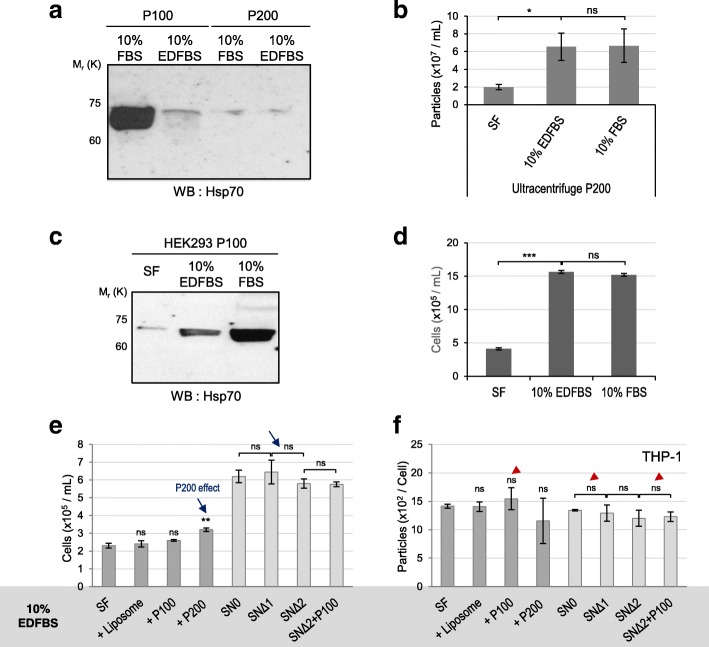
Exosome depleted FBS promotes cell proliferation but not exosome production. **a** Western blot of the P100 and P200 fractions isolated from the fresh 10% FBS and 10% ED-FBS media for detecting Hsp70. **b** The number of particles in the P200 fractions in different media: SF, 10% ED-FBS, 10% FBS. **c** The P100 fractions isolated from HEK293 CM under different culture conditions: SF, 10% FBS, 10% ED-FBS. The levels of Hsp70 are shown. **d** The cell number of HEK293 cells cultured in different media: SF, 10% FBS, 10% ED-FBS. **e, f** The fresh SF media, SF + liposomes, SF + P100 and SF + P200 fractions prepared from 10% ED-FBS media were supplied to the same number of THP-1 cells (dark gray). SN_0_, SN_Δ1,_ SN_Δ2_ and SN_Δ2_ + P100 fractions prepared from the 10% ED-FBS media were supplied to THP-1 cells (light gray). (**e**) The final cell numbers of THP-1 cell culture, and (**f**) the number of exosomes normalized by the final cell numbers. Blue arrows indicate marked effects by the P200 addition and red arrowheads indicate insignificant changes by the P100 addition. Experiments were repeated three or six times, each including six technical repeats. The results are displayed as mean ± S.E.M. *Significant difference analyzed by t-test (*p < 0.05,**p < 0.01,***p < 0.001). ns = not significant. Full original blots are presented in Additional file 8: Figure S8f, g

We have so far shown that extracellular exosomes promote exosome production and the smaller EVs support cell proliferation. To confirm our findings, we used ED-FBS in cell culture and examined its effects on exosome production and cell proliferation. The number of exosomes in fresh 10% ED-FBS media was indeed far less than fresh 10% FBS media (Additional file 4: Figure S4d; compare shaded blue and red). Accordingly, the P100 fraction prepared from 10% ED-FBS had significantly lower level of Hsp70 than those prepared from 10% FBS, while the P200 fractions prepared from these two sources showed comparable levels of Hsp70 (Fig. 6a, Additional file 8: Figure S8f). The number of particles in P200 fraction of 10% ED-FBS was also comparable to that of 10% FBS (Fig. 6b). Therefore, we confirmed that the P100 (exosomes) but not P200 (small EVs) is depleted in the ED-FBS.

When HEK293 cells were cultured in 10% ED-FBS medium or SF condition, similar number of exosomes was produced (Additional file 4: Figure S4d; compare gray and blue solid bars). The level of Hsp70 was also much lower when cells were cultured in SF or 10% ED-FBS than 10% FBS (Fig. 6c, Additional file 8: Figure S8g). On the contrary, the HEK293 cells exhibited robust cell proliferation in 10% ED-FBS media comparable to those in the 10% FBS media (Fig. 6d), indicating that ED-FBS is as effective as FBS in supporting cell proliferation (Fig. 6d, Additional file 6: Figure S6e). This finding is consistent with our previous data that the small EVs in the P200 fraction enhance cell proliferative ability.

To further confirm the differential functions of these two types of EVs, we prepared ultracentrifugal fractions from 10% ED-FBS media and examined their effect on exosome production and cell proliferation in THP-1 and HEK293 cell cultures. As ED-FBS essentially lacks exosomes, effects of the P100 fraction are expected to be absent in ED-FBS. Accordingly, addition of neither P100 nor P200 fractions led to any significant changes in number of secreted exosomes, compared to the SF control, both for THP-1 (Fig. 6f, red arrowheads; Additional file 7: Figure S7 g) and HEK293 cells (Additional file 7: Figure S7a, b, e). For cell proliferation, the final cell numbers showed a mild but statistically significant increase by adding the P200 prepared from ED-FBS, indicating that the small EVs are present in ED-FBS (Fig. 6e; blue arrows). Therefore, ED-FBS lost the capacity for exosome production but maintained the capacity for cell proliferation because ED-FBS lacks exosomes but not the smaller EVs.

We also prepared fractions from the CM of HEK293 cells cultured in 10% ED-FBS that contains small number of exosomes (Additional file 4: Figure S4d), and examined the effects of those fractions in HEK293 cell culture. As expected, the positive effect of the P200 fraction on cell proliferation was observed (Additional file 7: Figure S7c). Unlike the P100 fraction from fresh 10% ED-FBS, however, exosome production was enhanced by P100 from CM of HEK293 cells (10% ED-FBS) probably due to its higher number of exosome contents than the fresh 10% ED-FBS (Additional file 7: Figure S7d, f). Altogether, these data demonstrate that exosomes further promote exosome production and the smaller EVs enhance cell proliferation.

## Discussions

Studies on EVs recently have become increasingly active, and there are compelling evidences that cells secrete different populations of EVs generated by multiple intracellular mechanisms [45, 46]. We report here a novel type of EVs that are smaller than exosomes. They are isolated from HEK293 cell conditioned media or FBS, and are distinguished from exosomes in multiple ways. First, the sizes of the P200 vesicles (small EVs) are smaller than P100 vesicles (exosomes), shown by electron microscopy and NTA. The CD81 exosome marker is not detected in the smaller EVs while other exosome markers such as Hsp70, TSG101, and CD63 are present in both vesicle types. In addition, the smaller EVs are generated by a different biogenesis pathway, which is largely independent from the endocytic pathway by which the majority of exosomes are released. Ultimately, exosomes and the smaller EVs are functionally distinct, as extracellular exosomes promote exosome production while the smaller EVs support cell proliferation in human tissue culture. These smaller EVs are secreted by human, bovine and fly cells, implying their function in cell proliferation may be evolutionarily conserved.

Differential roles of exosomes and the smaller EVs were clearly demonstrated by addition or depletion of the P100 and P200 fractions in SF media for cell culture (Figs. 4, 5). It has been reported that removal of EVs from FBS by centrifugation at 120,000×g decreases the rate of cell proliferation [47]. However, these studies do not separate smaller vesicle populations away from exosome fractions. We showed that depletion of P200 fraction significantly reduces cell proliferation (Fig. 5b, e, g), indicating that the P200 small EVs contain essential factors to stimulate cell proliferation. What are the key signaling molecules in the small EVs? Signaling ligands such as Wnt and Hh seem to be present in the small EVs [48, 49]. Wnt3A is present in P200 fraction prepared from primary cultured rat glia [48]. We also found that mammalian Wnt1, Wnt3A, Wnt4 and the fly Wg are present not only in the P100 but also in the P200 (unpublished data). Identification of the factors responsible for cell proliferation in smaller EVs is an essential research topic in the future.

What are the mechanisms involved in biogenesis of small EVs? We showed that the small EV secretion is unaffected by BIG2 and N-SMase inhibitor, suggesting that distinct yet unidentified mechanisms control secretion of these small EVs (Fig. 2). However, they are likely to have a shared or at least overlapping origin with exosomes as both EV types contain CD63, a representative MVB marker [50], on their membrane structure. The smaller EVs may be sorted into a separate secretion pathway from MVB. It would be interesting to identify the molecular pathway responsible for the P200 small EV secretion. In addition, the regulatory mechanisms controlling EV secretion, for both exosomes and the small EVs, are largely unknown. Our study hinted that exosome itself harbors a clue to understand how its secretion can be regulated.

In this study, we showed that the P100 exosome and P200 smaller EVs support exosome production and cell proliferation, respectively. We expect these two types of EVs may be broadly useful in biomedical applications. For example, embryonic stem cell-derived exosomes are effective in cardiac regeneration, offering an alternative to stem cell therapy [4] and exosomes are even being developed for carriers for drug delivery [51]. Moreover, P200 small EVs can be applied as supplement for enhancing cell proliferation in industrial bioreactors. Further studies on their molecular signature, specific functions, and medical and industrial applications of these small EVs are warranted.

## Conclusions

Our study for the first time identifies the small EVs that are isolated by a subsequent ultracentrifugation of the supernatant fraction after the conventional exosome separation. This EV population is distinguished from exosomes in its smaller size, the protein contents, and the major biogenesis mechanisms. Furthermore, these small EVs support cell proliferation whereas exosomes promote additional exosome production. In sum, we report a new group of EVs that are distinct physically, biologically and functionally from exosomes. Our findings open up a new avenue to discover molecular information carried by EVs. Elucidating molecular signatures such as proteins and microRNAs carried by exosomes and the smaller EVs and their functions in recipient cells will greatly help our understanding on these fascinating vesicles.

## Additional files

Additional file 1: **Figure S1.** Vesicles in the P200 fractions from multiple cell lines are smaller than exosomes. (**a-a”’**) Representative TEM images of vesicles in (**a**) PBS, (**a’**) SN_Δ1_, (**a”**) SN_Δ2_ and (**a”’**) P200 fraction from the SN_Δ2_ of HEK293 CM. (**b-b”’**) Representative ESEM images of vesicles in (**b**) PBS, (**b’**) SN_Δ1_, (**b”**) SN_Δ2_ and (**b”’**) P200 fraction from the SN_Δ2_ of HEK293 CM. (**c**,**c’**) Representative TEM images of the vesicles in (**c**) P100 and (**c’**) P200 fraction from the S2 cell line. (**d**) Histograms of the particle size distribution in the P100 (blue) and P200 (red) fractions from TEM images of S2 CM (*n* = 500). (**e**) The bar graphs show the number of exosomes isolated from 10% FBS (blue) and HEK293 cell CM (light blue) after BFA, GW4869 treatment and control. The number of particles per mL in each ultracentrifugal sub-fractions from HEK293 cells cultured in SF media. (**f, g**) The bar graphs show (**f**) the number and (**g**) sizes of exosomes isolated from 10% FBS media (blue) and HEK293 cell CM (light blue) after BFA, GW4869 treatment and control. Experiments were repeated three times, each including six technical repeats. The results are displayed as mean ± S.E.M. Significant difference is tested by t-test (*p* > 0.05, ns = not significant). Scale bar: 100 nm. (PDF 424 kb)

Additional file 2: **Figure S2.** Multiple exosome marker analyses in exosome fractions obtained by ultracentrifugation and qEV column. (**a**) This plot depicts concentrations of total proteins in qEV fractions from HEK293 CM, measured by Bradford assay. (**b**,**c**) Western analysis of the qEV fractions for (**b**) Hsp70 and (**c**) CD63. (**d**,**e**) Western analysis of the P100 and P200 fractions in 10% FBS THP1 CM by anti-CD81 antibody (SBI). (**f**,**g**) Western blots using anti-CD81 antibody using qEV fractions isolated from HEK293 cells cultured in (**f**) SF media for 24 h and (**g**) 10% FBS media for 72 h. Red arrow indicates the weak CD81 signal. Full-length original blots are presented in Additional file 9: Figure S9. (**h, i**) Representative TEM images of the vesicles in (**h**) P100 and (**i**) P200 stained with an anti-rabbit gold conjugated antibody for negative control (no primary antibody). Scale bar: 100 nm. (PDF 297 kb)

Additional file 3: **Figure S3.** Exosomes and the smaller EVs from HEK293 cells do not affect S2 cells. (**a-d**) These bar graphs show the number of exosomes per mL media about (**a**) Fig. 4c, (**b**) Fig. 4d, (**c**) Fig. 5c, and (**d**) Fig. 5d. Red arrows indicate the P100 effects. (**e-h**) These bar graphs show the number of exosomes per mL media about (**e**) Fig. 4f, (**f**) Fig. 5f, (**g**) Fig. 4h and (**h**) Fig. 5h. Red arrows indicate the P100 effects. (**i-l**) The P100 fraction and P200 fraction isolated from HEK293 CM with control AS (artificial serum) were treated to *Drosophila* S2 cells. (**i**) Final number of cells, (**j**) number of exosomes per mL media, (**k**) number of exosomes per cell, (**l**) and cell viability (all comparisons: *p* > 0.05). Experiments were repeated three times, each including six technical repeats. The results are displayed as mean ± S.E.M. *Significant difference analyzed by t-test (*p < 0.05,**p < 0.01,***p < 0.001). ^+^ *p* < 0.1 and ns = not significant. SF = serum-free. (PDF 112 kb)

Additional file 4: **Figure S4.** FBS enhances exosome production. (**a**) Fold changes in number of particles per mL media and per final cell count in the P100 fractions when HEK293 and THP-1 cells were cultured in 10% FBS compared to SF conditions for 48 h. (**b**,**c**) These bar graphs show the number of exosomes (**b**) per mL media and (**c**) per final cell count when P100 exosomes were isolated from HEK293 CM grown in 10% FBS (blue) and in SF (red) for 48 h. (**d**) The number of particles per mL media were measured before (shaded) and 48 h after (solid) supplied to HEK293 cells. Exosome samples were isolated by ultracentrifugation (P100). Media were prepared in three types: SF (gray), 10% ED-FBS (exosome depleted FBS) (blue), and 10% FBS (red). (**e**,**f**) Time-series analysis of extracellular exosomes of (**e**) THP-1 cells and (**f**) HEK293 cells in either SF media (blue), SF media supplemented with the liposomes (orange), and P100 extracted from FBS (red). (**g**) Histograms showing the size distribution and concentrations of liposomes. (**h**) The graph shows the number of particles per mL in each ultracentrifugal sub-fraction from 10% FBS media. The exosomes were isolated by Exo-quick-TC. Experiments were repeated three times, each including six technical repeats. The results are displayed as mean ± S.E.M. *Significant difference analyzed by t-test (**p < 0.01,***p < 0.001). The dashed lines generated to estimate the time-series trend. SF = serum-free. (PDF 222 kb)

Additional file 5: **Figure S5.** Addition of FBS enhances exosome production of HEK293 cells. (**a-f**) These bar graphs show (**a**) the number of cells when HEK293 cells were cultured in the media containing indicated concentrations of FBS for 48 h, (**b**) the number of exosomes present in differentially diluted FBS before adding to the cells (EV_0h_), the number of exosomes (**c**) per mL CM (EV_48h_) and (**e**) per final cell count. The number of exosomes in FBS in (**b**) was subtracted from the number of exosomes in HEK293 CM to obtain the net increase in exosome number per mL media (**d**) or per cell number (**f**). (**g**) This graph shows the number of P200 vesicles when HEK293 cells were provided with liposomes, P200 vesicles, and three times concentrated P200 vesicles that had been prepared from HEK293 CM. Experiments were repeated three times, each including three technical repeats. The results are displayed as mean ± S.E.M. Significant difference analyzed by t-test (*p* > 0.05, ns = not significant). (PDF 43 kb)

Additional file 6: **Figure S6.** Cell viability results associated with Figs. 4, 5, 6. (**a-d**) Cell viability of HEK293 or THP-1 cells 48 h after supplied with indicated ultracentrifugal sub-fractions isolated from (**a**,**c**) 10% FBS media or (**b**,**d**) HEK293 CM. (**e**) The graph shows the cell viability of HEK293 cells cultured in each medium: SF, 10% ED-FBS, 10% FBS in Fig. 6d. Value = mean ± S.E.M. (PDF 37 kb)

Additional file 7: **Figure S7.** 10% ED-FBS HEK293 CM promotes exosome production and cell proliferation. (**a**, **b**, **e**) When the liposome, P100, P200 fractions from 10% ED-FBS were added to SF media, and SN_0_, SN_Δ1,_ SN_Δ2_ and SN_Δ2_ + P100 fractions from the 10% ED-FBS culture media was supplied to HEK293 cells, (**a**) final cell numbers of HEK293, (**b**) the number of exosome normalized to the final cell numbers, and (**e**) the number of exosome per ml were counted. (**c**,**d**,**f**) When HEK293 cells were treated with the ultracentrifugal fractions prepared from 10% ED-FBS HEK293 CM, (**c**) the final cell numbers for HEK293 cells, (**d**) the number of exosomes normalized to the final cell numbers, and (**f**) the number of exosome per ml. (**g**) The number of exosome per ml in Fig. 6f. Red arrows indicate the P100 effect and blue arrows indicate the P200 effect. Red arrowheads mean not significant change by P100 fraction. Experiments were repeated three times, each including six technical repeats. The results are displayed as mean ± S.E.M. *Significant difference analyzed by t-test (*p < 0.05,**p < 0.01,***p < 0.001). ^+^ *p* < 0.1 and ns = not significant. SF = serum-free. (PDF 121 kb)

Additional file 8: **Figure S8.** Original full western blots associated with main figures. (**a-e**) The original western blots of Fig. 3a-e. (**f**,**g**) The original western blots of Fig. 6a, c. (PDF 98 kb)

Additional file 9: **Figure S9.** Original full western blots associated with supplementary figures. (**a-f**) The original western blots of Additional file 2: Figure S2 b-g. (PDF 123 kb)

## Data Availability

All data generated in this study are included in the manuscript.

## Abbreviations

FBS: Fetal bovine serum
SF: Serum free
EDFBS: Exosome-depleted fetal bovine serum
EV: Extracellular vesicle
