# Supplementary figures and images for: A novel population of extracellular vesicles smaller than exosomes promotes cell proliferation
Source: Cell Commun Signal. 2019 Aug 15;17:95. doi: 10.1186/s12964-019-0401-z (PMC6694590; doi:10.1186/s12964-019-0401-z)

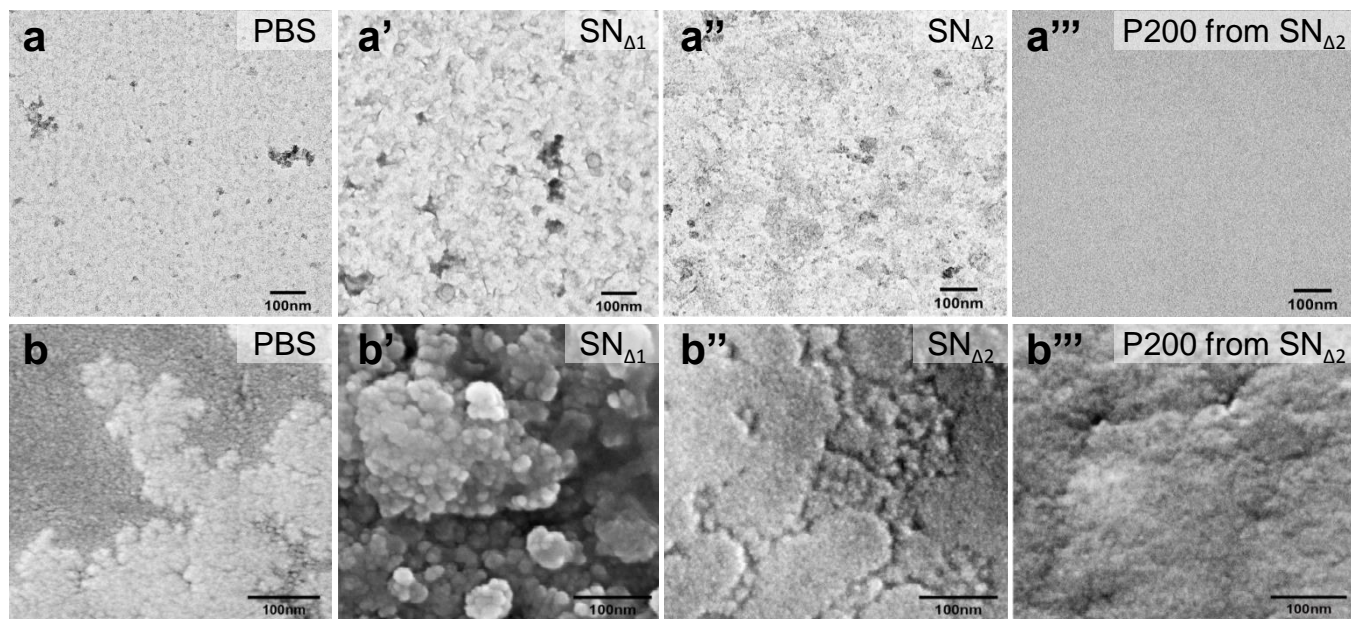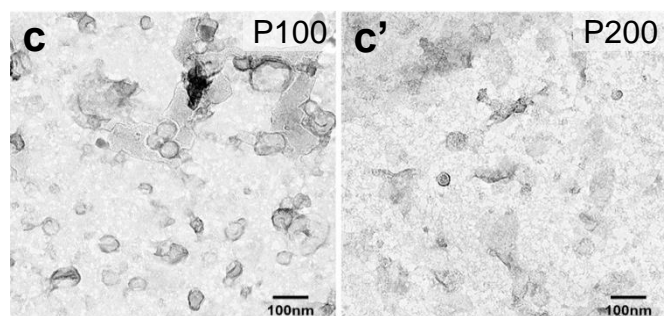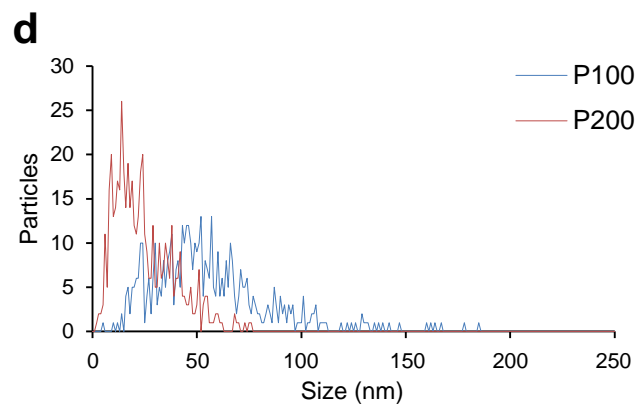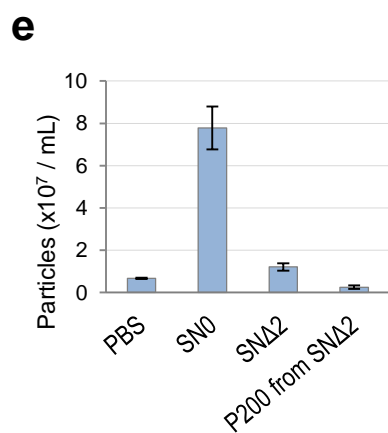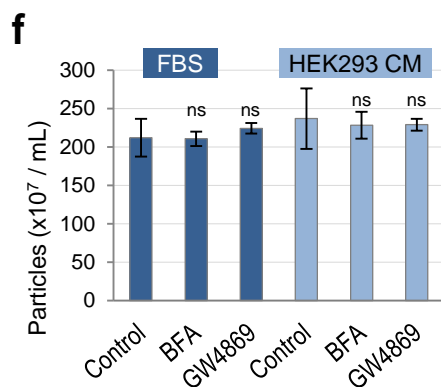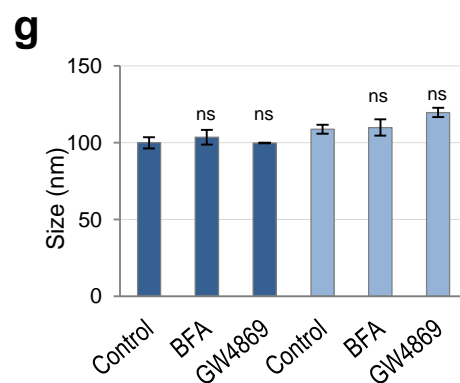

Supplement: Supplementary file 1 — Figure S1. Vesicles in the P200 fractions from multiple cell lines are smaller than exosomes. (a-a”’) Representative TEM images of vesicles in (a) PBS, (a’) SNΔ1, (a”) SNΔ2 and (a”’) P200 fraction from the SNΔ2 of HEK293 CM. (b-b”’) Representative ESEM images of vesicles in (b) PBS, (b’) SNΔ1, (b”) SNΔ2 and (b”’) P200 fraction from the SNΔ2 of HEK293 CM. (c,c’) Representative TEM images of the vesicles in (c) P100 and (c’) P200 fraction from the S2 cell line. (d) Histograms of the particle size distribution in the P100 (blue) and P200 (red) fractions from TEM images of S2 CM (n = 500). (e) The bar graphs show the number of exosomes isolated from 10% FBS (blue) and HEK293 cell CM (light blue) after BFA, GW4869 treatment and control. The number of particles per mL in each ultracentrifugal sub-fractions from HEK293 cells cultured in SF media. (f, g) The bar graphs show (f) the number and (g) sizes of exosomes isolated from 10% FBS media (blue) and HEK293 cell CM (light blue) after BFA, GW4869 treatment and control. Experiments were repeated three times, each including six technical repeats. The results are displayed as mean ± S.E.M. Significant difference is tested by t-test (p > 0.05, ns = not significant). Scale bar: 100 nm. (PDF 424 kb) [file 12964_2019_401_MOESM1_ESM.pdf]

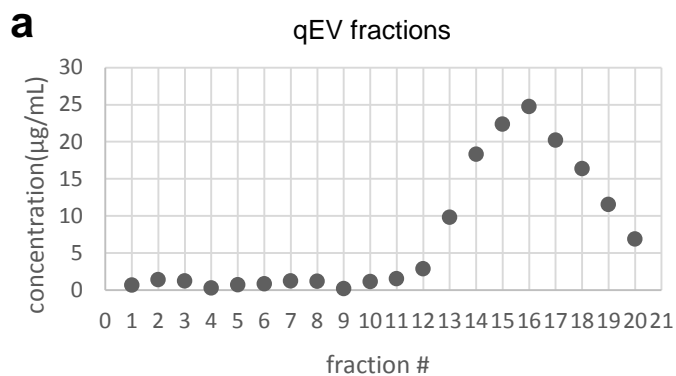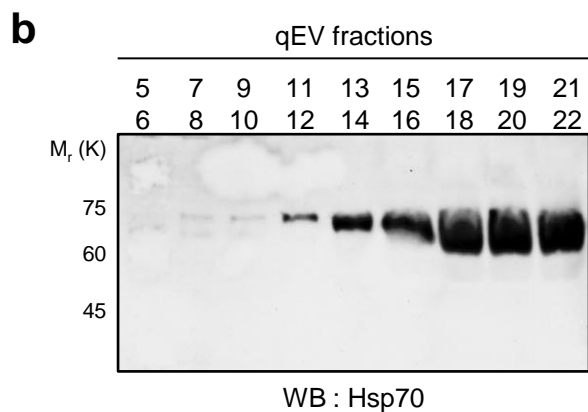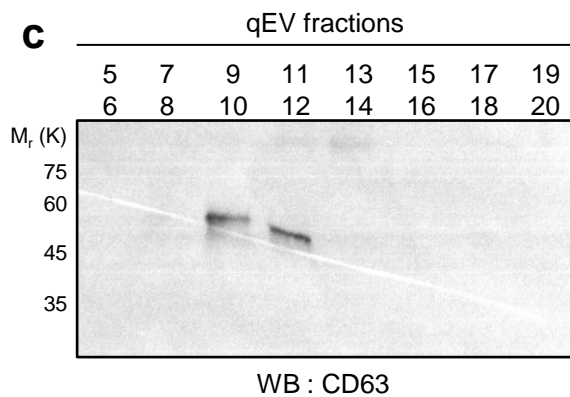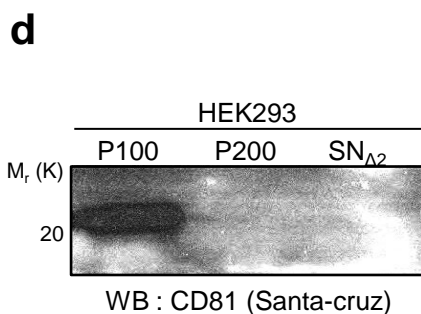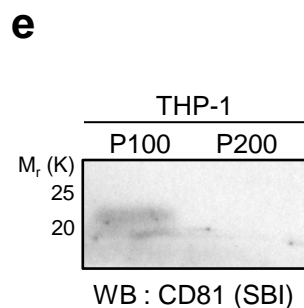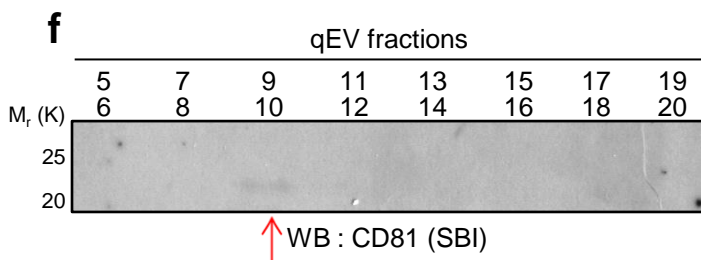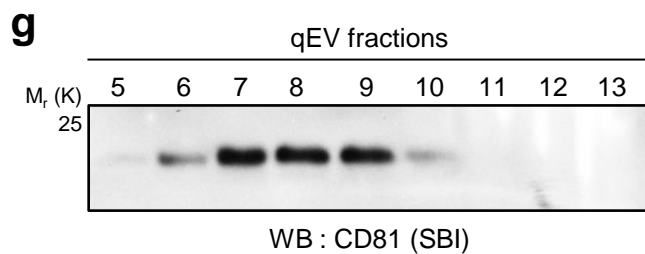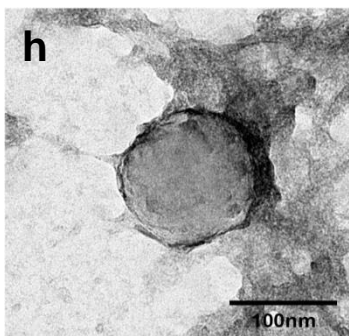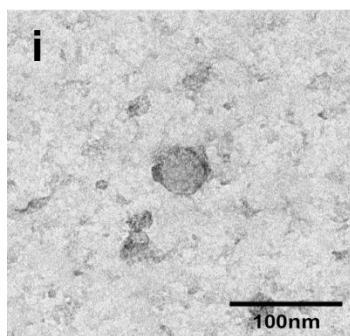

Supplement: Supplementary file 2 — Figure S2. Multiple exosome marker analyses in exosome fractions obtained by ultracentrifugation and qEV column. (a) This plot depicts concentrations of total proteins in qEV fractions from HEK293 CM, measured by Bradford assay. (b,c) Western analysis of the qEV fractions for (b) Hsp70 and (c) CD63. (d,e) Western analysis of the P100 and P200 fractions in 10% FBS THP1 CM by anti-CD81 antibody (SBI). (f,g) Western blots using anti-CD81 antibody using qEV fractions isolated from HEK293 cells cultured in (f) SF media for 24 h and (g) 10% FBS media for 72 h. Red arrow indicates the weak CD81 signal. Full-length original blots are presented in Additional file 9: Figure S9. (h, i) Representative TEM images of the vesicles in (h) P100 and (i) P200 stained with an anti-rabbit gold conjugated antibody for negative control (no primary antibody). Scale bar: 100 nm. (PDF 297 kb) [file 12964_2019_401_MOESM2_ESM.pdf]

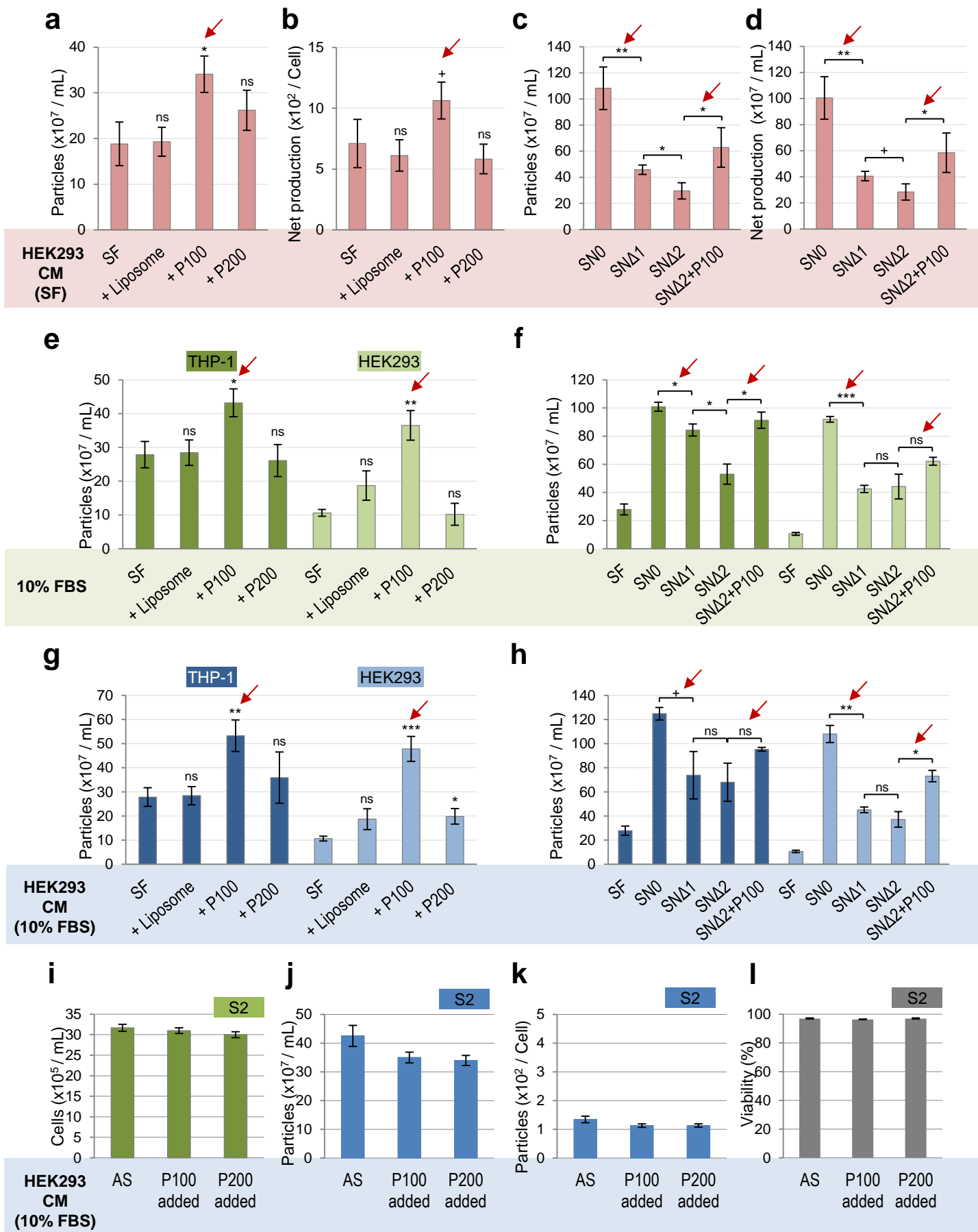

Supplement: Supplementary file 3 — Figure S3. Exosomes and the smaller EVs from HEK293 cells do not affect S2 cells. (a-d) These bar graphs show the number of exosomes per mL media about (a) Fig. 4c, (b) Fig. 4d, (c) Fig. 5c, and (d) Fig. 5d. Red arrows indicate the P100 effects. (e-h) These bar graphs show the number of exosomes per mL media about (e) Fig. 4f, (f) Fig. 5f, (g) Fig. 4h and (h) Fig. 5h. Red arrows indicate the P100 effects. (i-l) The P100 fraction and P200 fraction isolated from HEK293 CM with control AS (artificial serum) were treated to Drosophila S2 cells. (i) Final number of cells, (j) number of exosomes per mL media, (k) number of exosomes per cell, (l) and cell viability (all comparisons: p > 0.05). Experiments were repeated three times, each including six technical repeats. The results are displayed as mean ± S.E.M. *Significant difference analyzed by t-test (*p < 0.05,**p < 0.01,***p < 0.001). + p < 0.1 and ns = not significant. SF = serum-free. (PDF 112 kb) [file 12964_2019_401_MOESM3_ESM.pdf]

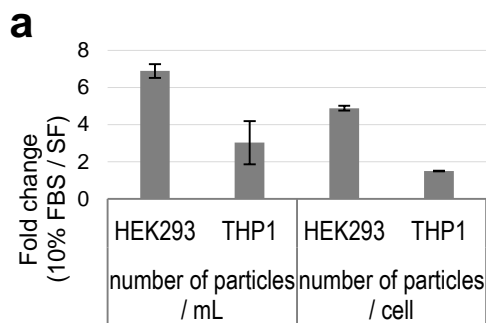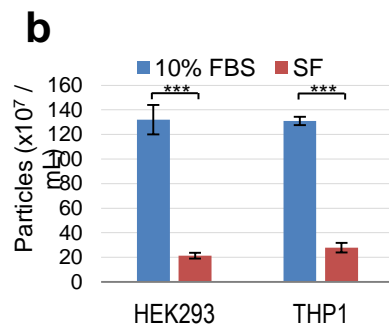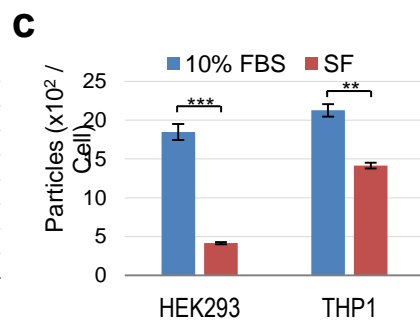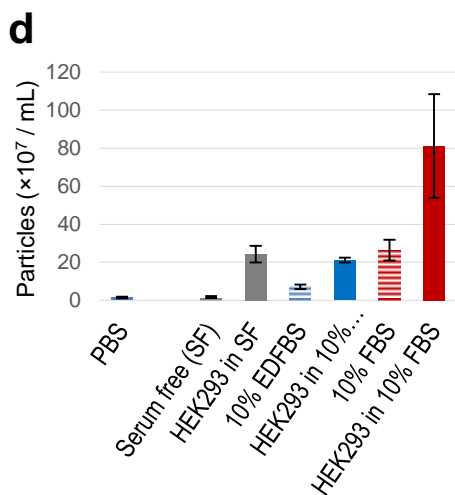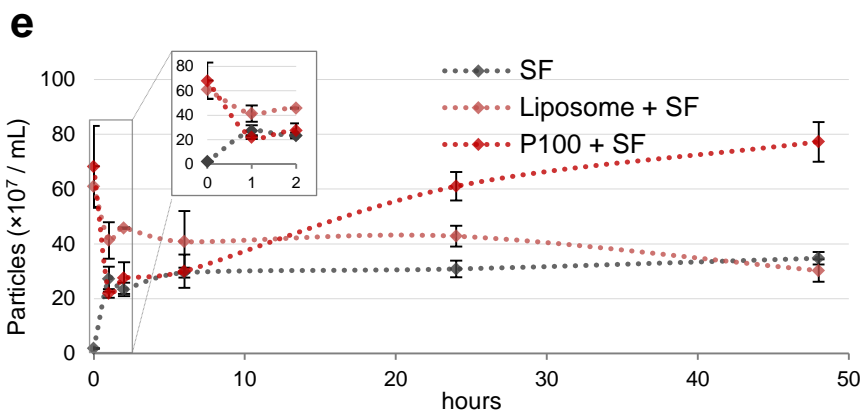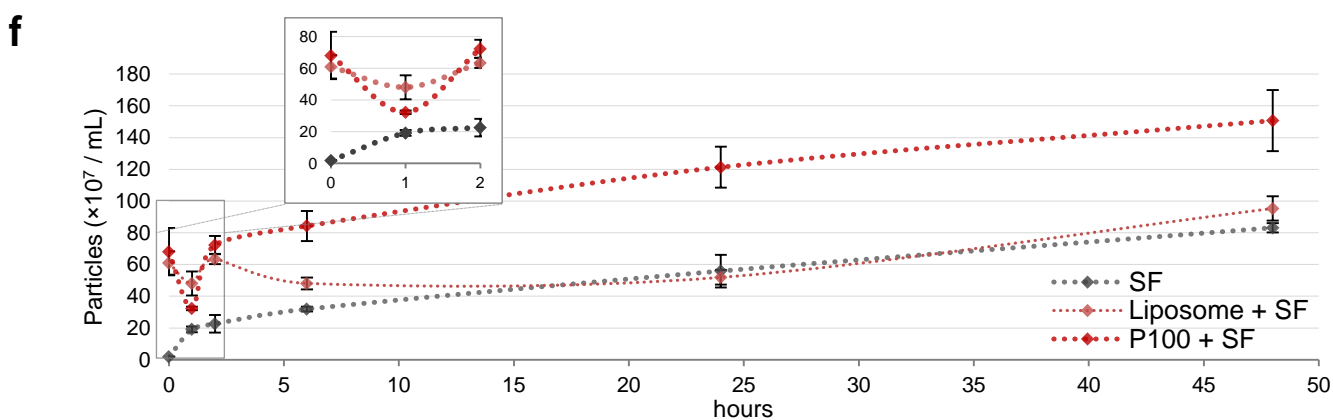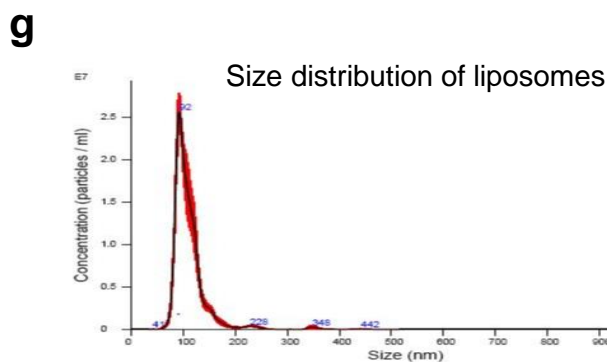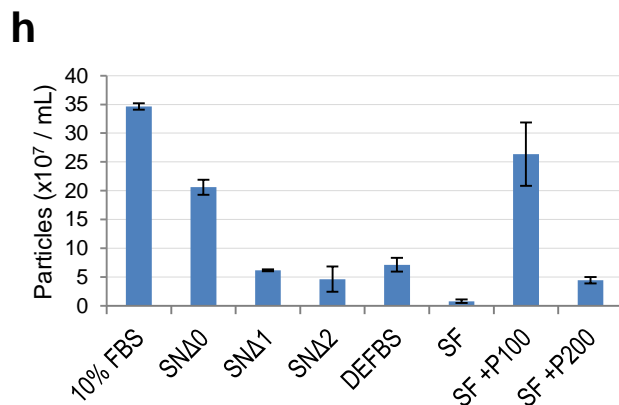

Supplement: Supplementary file 4 — Figure S4. FBS enhances exosome production. (a) Fold changes in number of particles per mL media and per final cell count in the P100 fractions when HEK293 and THP-1 cells were cultured in 10% FBS compared to SF conditions for 48 h. (b,c) These bar graphs show the number of exosomes (b) per mL media and (c) per final cell count when P100 exosomes were isolated from HEK293 CM grown in 10% FBS (blue) and in SF (red) for 48 h. (d) The number of particles per mL media were measured before (shaded) and 48 h after (solid) supplied to HEK293 cells. Exosome samples were isolated by ultracentrifugation (P100). Media were prepared in three types: SF (gray), 10% ED-FBS (exosome depleted FBS) (blue), and 10% FBS (red). (e,f) Time-series analysis of extracellular exosomes of (e) THP-1 cells and (f) HEK293 cells in either SF media (blue), SF media supplemented with the liposomes (orange), and P100 extracted from FBS (red). (g) Histograms showing the size distribution and concentrations of liposomes. (h) The graph shows the number of particles per mL in each ultracentrifugal sub-fraction from 10% FBS media. The exosomes were isolated by Exo-quick-TC. Experiments were repeated three times, each including six technical repeats. The results are displayed as mean ± S.E.M. *Significant difference analyzed by t-test (**p < 0.01,***p < 0.001). The dashed lines generated to estimate the time-series trend. SF = serum-free. (PDF 222 kb) [file 12964_2019_401_MOESM4_ESM.pdf]

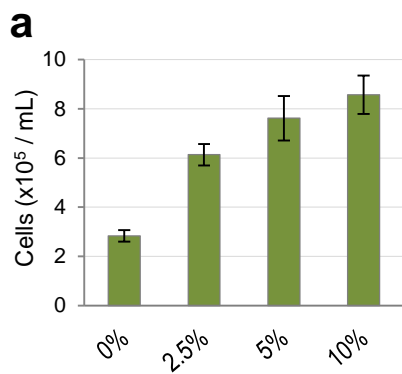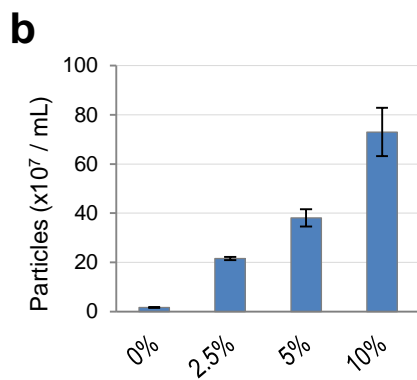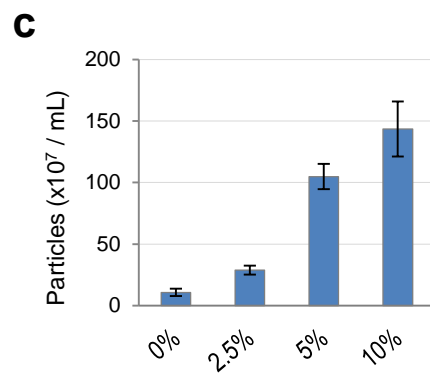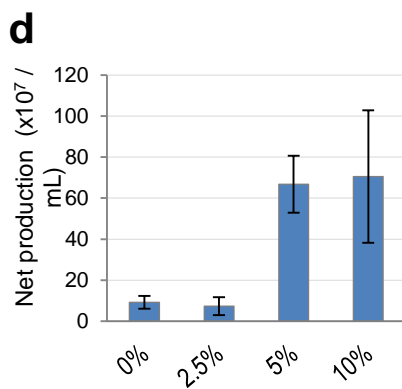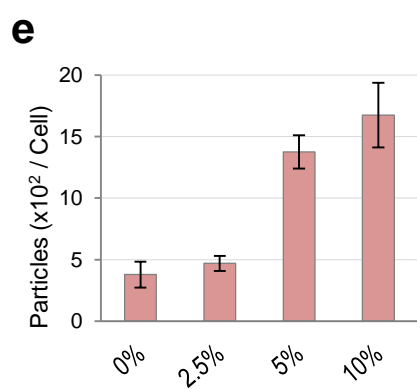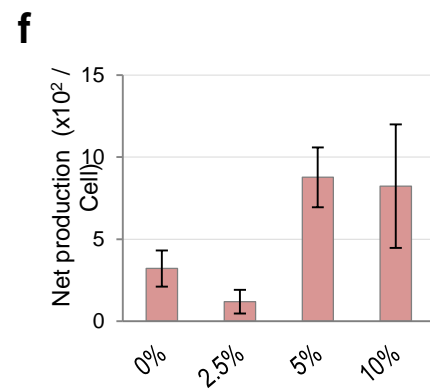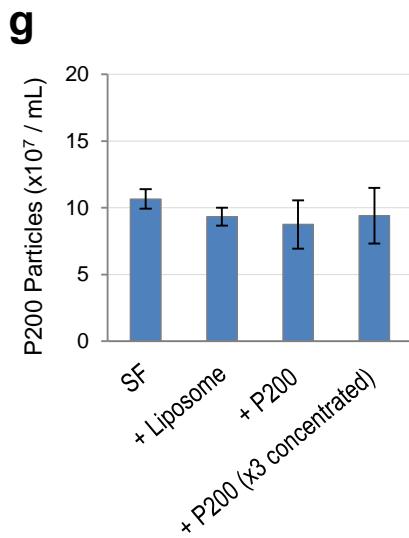

Supplement: Supplementary file 5 — Figure S5. Addition of FBS enhances exosome production of HEK293 cells. (a-f) These bar graphs show (a) the number of cells when HEK293 cells were cultured in the media containing indicated concentrations of FBS for 48 h, (b) the number of exosomes present in differentially diluted FBS before adding to the cells (EV0h), the number of exosomes (c) per mL CM (EV48h) and (e) per final cell count. The number of exosomes in FBS in (b) was subtracted from the number of exosomes in HEK293 CM to obtain the net increase in exosome number per mL media (d) or per cell number (f). (g) This graph shows the number of P200 vesicles when HEK293 cells were provided with liposomes, P200 vesicles, and three times concentrated P200 vesicles that had been prepared from HEK293 CM. Experiments were repeated three times, each including three technical repeats. The results are displayed as mean ± S.E.M. Significant difference analyzed by t-test (p > 0.05, ns = not significant). (PDF 43 kb) [file 12964_2019_401_MOESM5_ESM.pdf]

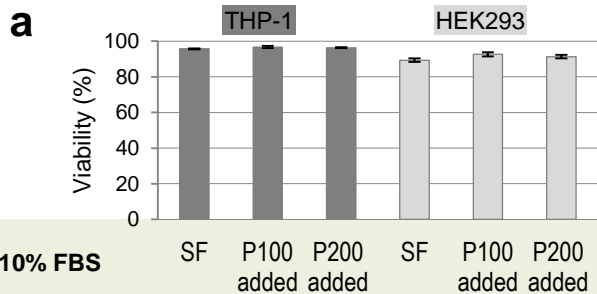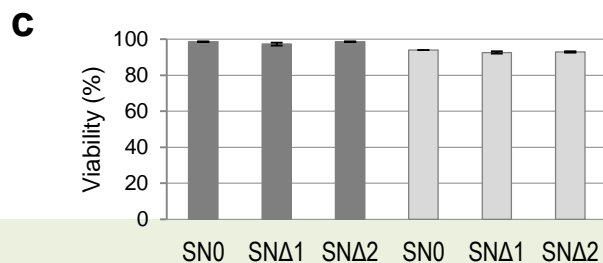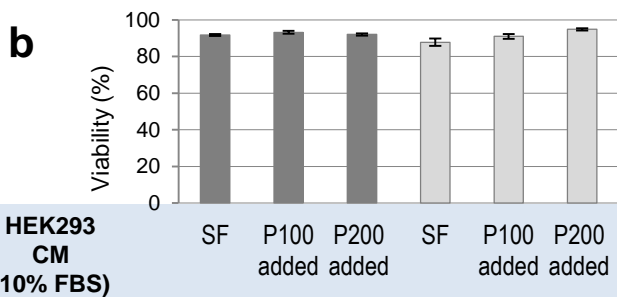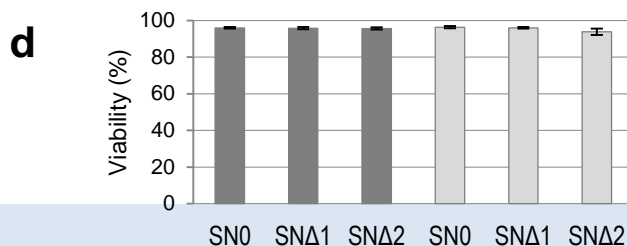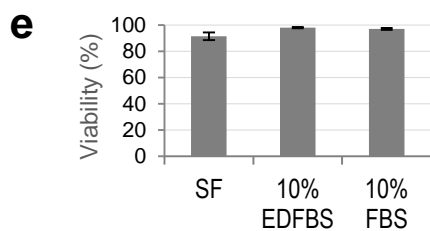

Supplement: Supplementary file 6 — Figure S6. Cell viability results associated with Figs. 4, 5, 6. (a-d) Cell viability of HEK293 or THP-1 cells 48 h after supplied with indicated ultracentrifugal sub-fractions isolated from (a,c) 10% FBS media or (b,d) HEK293 CM. (e) The graph shows the cell viability of HEK293 cells cultured in each medium: SF, 10% ED-FBS, 10% FBS in Fig. 6d. Value = mean ± S.E.M. (PDF 37 kb) [file 12964_2019_401_MOESM6_ESM.pdf]

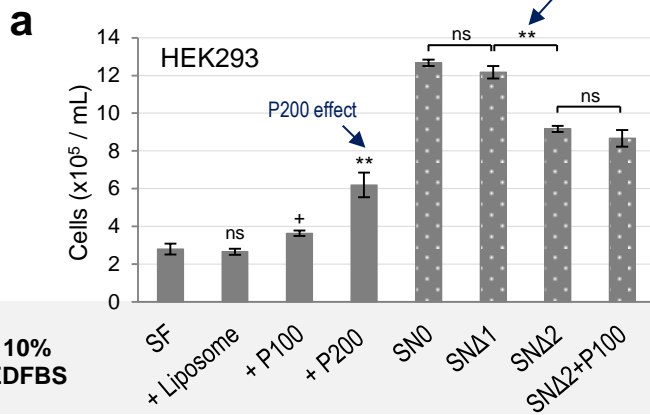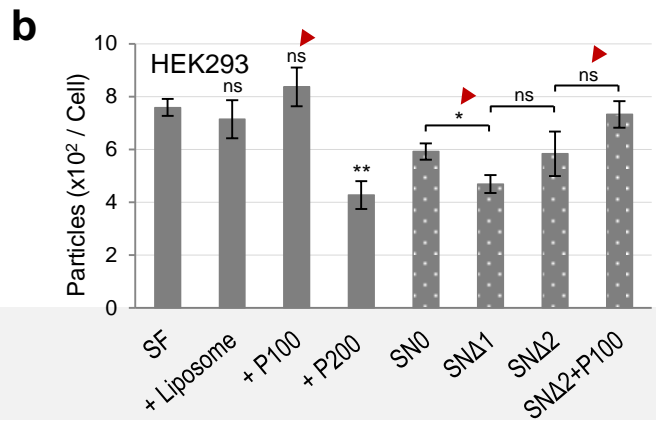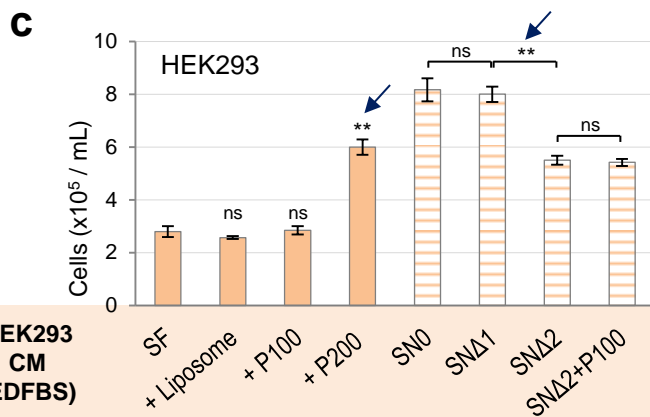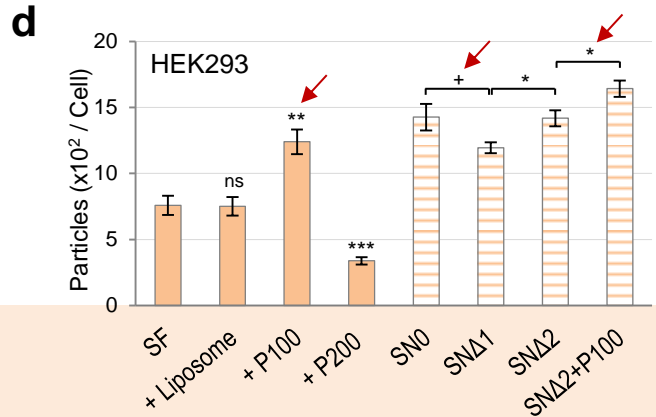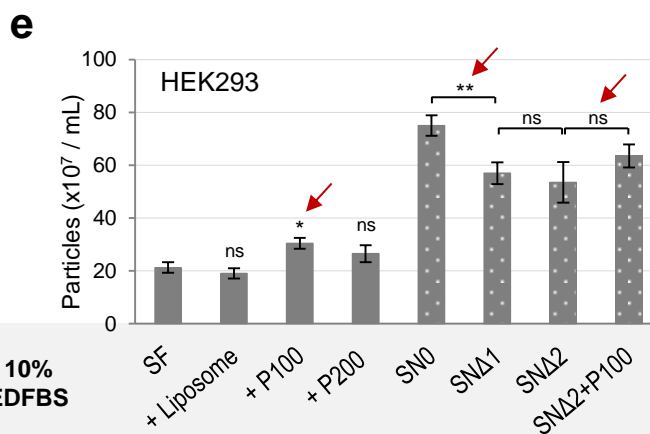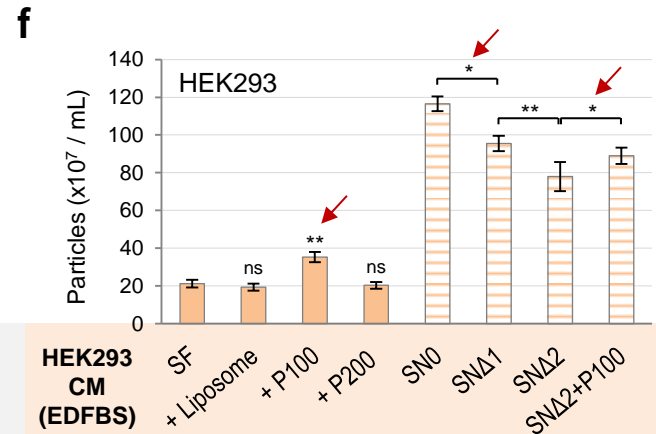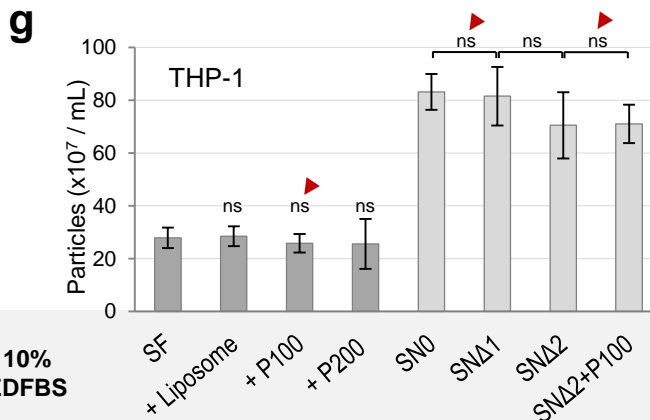

Supplement: Supplementary file 7 — Figure S7. 10% ED-FBS HEK293 CM promotes exosome production and cell proliferation. (a, b, e) When the liposome, P100, P200 fractions from 10% ED-FBS were added to SF media, and SN0, SNΔ1, SNΔ2 and SNΔ2 + P100 fractions from the 10% ED-FBS culture media was supplied to HEK293 cells, (a) final cell numbers of HEK293, (b) the number of exosome normalized to the final cell numbers, and (e) the number of exosome per ml were counted. (c,d,f) When HEK293 cells were treated with the ultracentrifugal fractions prepared from 10% ED-FBS HEK293 CM, (c) the final cell numbers for HEK293 cells, (d) the number of exosomes normalized to the final cell numbers, and (f) the number of exosome per ml. (g) The number of exosome per ml in Fig. 6f. Red arrows indicate the P100 effect and blue arrows indicate the P200 effect. Red arrowheads mean not significant change by P100 fraction. Experiments were repeated three times, each including six technical repeats. The results are displayed as mean ± S.E.M. *Significant difference analyzed by t-test (*p < 0.05,**p < 0.01,***p < 0.001). + p < 0.1 and ns = not significant. SF = serum-free. (PDF 121 kb) [file 12964_2019_401_MOESM7_ESM.pdf]

**a**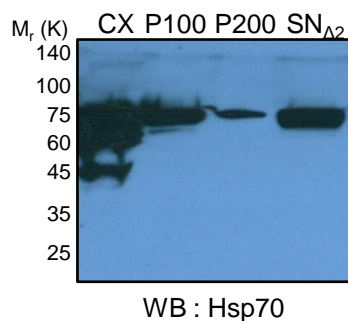**b**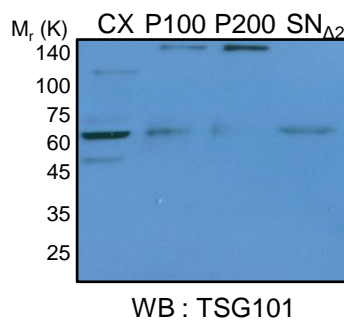**c**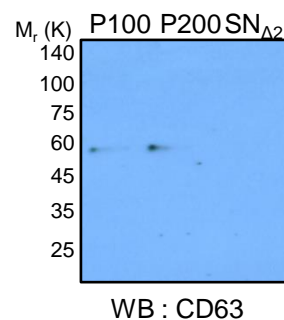**d**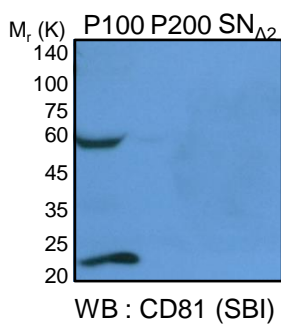**e**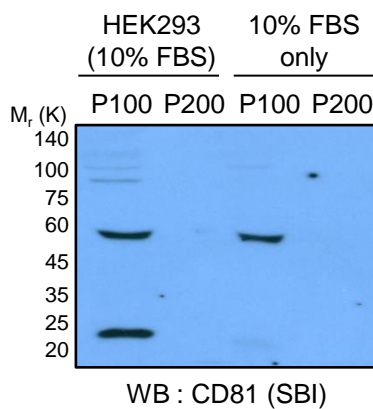**f**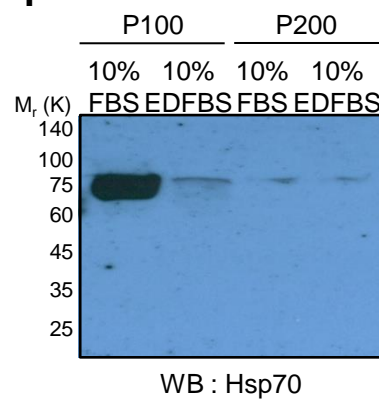**g**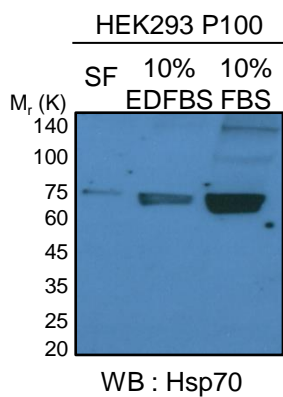

Supplement: Supplementary file 8 — Figure S8. Original full western blots associated with main figures. (a-e) The original western blots of Fig. 3a-e. (f,g) The original western blots of Fig. 6a, c. (PDF 98 kb) [file 12964_2019_401_MOESM8_ESM.pdf]

**a**

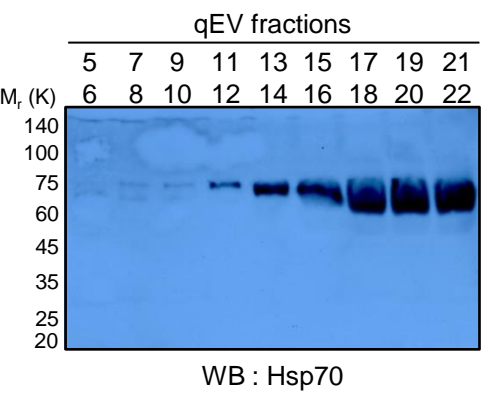

**b**

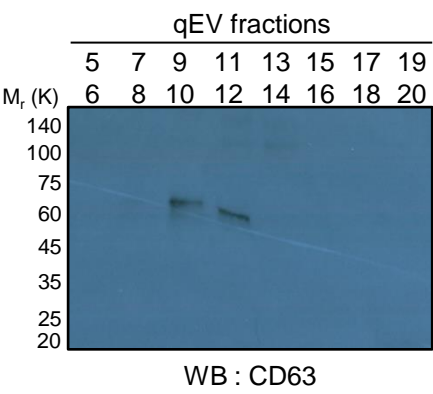

**c**

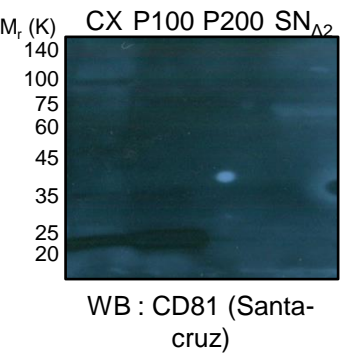

**d**

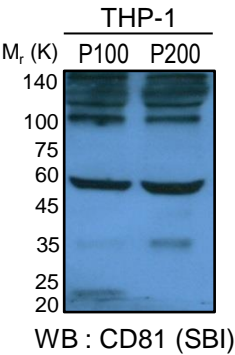

**e**

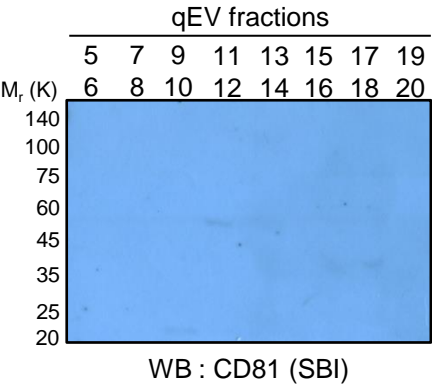

**f**

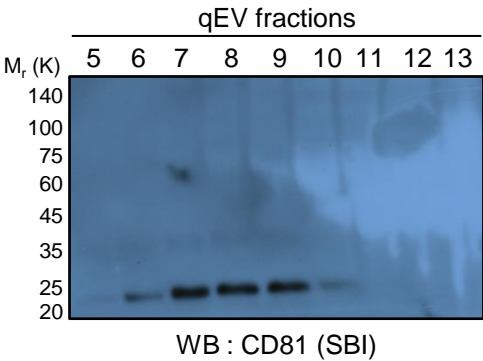

Supplement: Supplementary file 9 — Figure S9. Original full western blots associated with supplementary figures. (a-f) The original western blots of Additional file 2: Figure S2 b-g. (PDF 123 kb) [file 12964_2019_401_MOESM9_ESM.pdf]
